# Competitive Redox Chemistries in Vanadium Niobium Oxide for Ultrafast and Durable Lithium Storage

**DOI:** 10.1007/s40820-023-01172-9

**Published:** 2023-08-10

**Authors:** Xiaobo Ding, Jianhao Lin, Huiying Huang, Bote Zhao, Xunhui Xiong

**Affiliations:** School of Environment and Energy, Guangdong Provincial Key Laboratory of Advanced Energy Storage Materials, South China University of Technology, Guangzhou, 510640 People’s Republic of China

**Keywords:** Niobium pentoxide, Capacity decay, Over-reduction, Vanadium niobium oxide, Lithium-ion capacitor

## Abstract

Niobium pentoxide (Nb_2_O_5_) anodes have gained increasing attentions for high-power lithium-ion batteries owing to the outstanding rate capability and high safety. However, Nb_2_O_5_ anode suffers poor cycle stability even after modified and the unrevealed mechanisms have restricted the practical applications. Herein, the over-reduction of Nb^5+^ has been demonstrated to be the critical reason for the capacity loss for the first time. Besides, an effective competitive redox strategy has been developed to solve the rapid capacity decay of Nb_2_O_5_, which can be achieved by the incorporation of vanadium to form a new rutile VNbO_4_ anode. The highly reversible V^3+^/V^2+^ redox couple in VNbO_4_ can effectively inhibit the over-reduction of Nb^5+^. Besides, the electron migration from V^3+^ to Nb^5+^ can greatly increase the intrinsic electronic conductivity for VNbO_4_. As a result, VNbO_4_ anode delivers a high capacity of 206.1 mAh g^−1^ at 0.1 A g^−1^, as well as remarkable cycle performance with a retention of 93.4% after 2000 cycles at 1.0 A g^−1^. In addition, the assembled lithium-ion capacitor demonstrates a high energy density of 44 Wh kg^−1^ at 5.8 kW kg^−1^. In summary, our work provides a new insight into the design of ultra-fast and durable anodes.

**Supplementary Information:**

The online version contains supplementary material available at 10.1007/s40820-023-01172-9.

## Highlights

- The over-reduction from Nb^5+^ to Nb^3+^ in the lithiation process have been demonstrated to be the critical reason for the capacity decay of Nb_2_O_5_ for the first time.
- A novel competitive redox strategy has been proposed to suppress the over-reduction of Nb^5+^ to Nb^3+^, which can be achieved by the incorporation of vanadium to form a new rutile VNbO_4_ anode.
- The performance of VNbO_4_ anode designed in this study stands among the best in cycle stability.

**Supplementary Information:**

The online version contains supplementary material available at 10.1007/s40820-023-01172-9.

## Graphical abstract

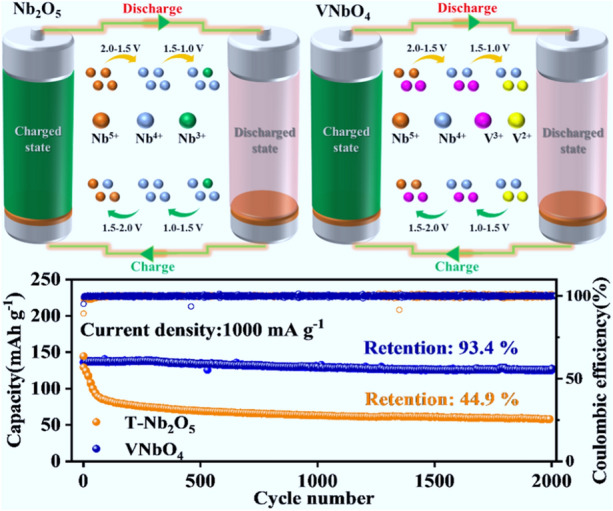

## Introduction

With the explosive growth of electric vehicles in the recent years, the inadequate power density and safety concerns have become the pivotal challenges of lithium-ion batteries (LIBs) and sodium-ion batteries (SIBs) for their further developments [1, 2]. However, the state-of-the-art graphite-based anodes cannot meet these critical demands because the low working potentials at 0.2 V can easily result in the generation of Li dendrites at high charge current densities [3–5]. In addition, the formation of a thick SEI film on surface of graphite anode at such low potential window hinders the fast transport of Li ions and increases heat amount, which will accelerate the deterioration of battery performance [6]. Thereafter, Li_4_Ti_5_O_12_ (LTO) and TiO_2_(B) anodes have been widely investigated as fast-charging electrodes owing to their fast Li^+^ insertion mechanism at ~ 1.55 V (vs. Li^+^/Li) [7, 8]. However, the rather low specific capacities severely restrict the applications in high-energy–density LIBs. Compared with Ti-based oxides, Nb_2_O_5_ shows a higher theoretical capacity owing to the rich redox chemistry of Nb element (Nb^5+^/Nb^4+^, Nb^4+^/Nb^3+^) in the potential range between 1.0 and 3.0 V versus Li^+^/Li as well as superior rate performances [9]. Besides, the electrolyte decomposition and the Li dendrite formation at anode side can be prevented, leading to a high safety for the Nb_2_O_5_-based batteries [10]. It is worth noting that the Nb_2_O_5_ exists in different polymorphs, including pseudohexagonal Nb_2_O_5_ (TT-Nb_2_O_5_), orthorhombic Nb_2_O_5_ (T-Nb_2_O_5_), tetragonal Nb_2_O_5_ (M-Nb_2_O_5_), and monoclinic Nb_2_O_5_ (H-Nb_2_O_5_), which show greatly different Li storage performances [11]. T-Nb_2_O_5_ possesses alternating loosely packed 4 g atomic layers and densely packed 4 h atomic layers in the *c*-axis direction, in which 4 g layer provides the space of inserted Li^+^ and 4 h layer provides coordination oxygen for Li^+^ diffusion with low steric hindrance, leading to excellent rate performances, while H-Nb_2_O_5_ with the Wadsley–Roth shear phase shows a flat potential plateau at ~ 1.65 V (vs. Li^+^/Li) owing to the two-phase transition process during the charge/discharge processes [12, 13]. Nevertheless, the poor electronic conductivity significantly restricts the applications of all the Nb_2_O_5_-based anodes for high-power LIBs [14].

Recently, various strategies have been developed to improve the rate performances of Nb_2_O_5_, including designing different Nb_2_O_5_ nanostructures, heteroatom doping and composing carbon matrix [15–17]. For example, Nb_2_O_5_ nanorod film grown directly on flexible carbon cloth showed a high Li storage capacity of 220 mAh g^−1^ at 0.5C and 160.6 mAh g^−1^ at 20C, respectively, which can be attributed to the remarkably decreased transfer distances of Li^+^/electrons in the charge–discharge processes [18]. *N* doping can greatly improve the electronic/ionic conductivities of Nb_2_O_5_, thus it exhibits high capacities of 202.9 and 104.6 mAh g^−1^ at current densities of 0.1C and 25C, respectively [19]. T-Nb_2_O_5_-carbon-graphene composite delivered a high capacity of 206 mAh g^−1^ at 0.5C and 114 mAh g^−1^ at 100C [20]. Despite of these impressive progresses in enhancing rate performances, almost all of the Nb_2_O_5_ anodes still showed fast capacity decay (Table S1), which has not yet been overcome in the previous works and the mechanisms are remaining unclear. For example, S-doped T-Nb_2_O_5_ encapsulated into S-doped graphene (S-Nb_2_O_5_@S-rGO) demonstrated a poor cycle stability from 325 to 215 mAh g^−1^ after 100 cycles at 0.5C [21]. Similar phenomenon is also observed in Nb_2_O_5 − *x*_ NP, which showed fast capacity deterioration from 245 to 175 mAh g^−1^ after 200 cycles at 0.2 A g^−1^ [22]. Therefore, the in-depth investigation of the fast capacity decay during the long-term cycles and the exploration of efficient strategies to address this challenge are urgently needed.

Herein, the over-reduction of Nb^5+^ into Nb^3+^ in the lithiation process and then the incomplete oxidation in the following delithiation process have been demonstrated to be the critical reason for the rapid capacity decrease in Nb_2_O_5_ during cycling. Based on the failure mechanism, vanadium has been proposed to incorporate into Nb_2_O_5_ to form a new rutile VNbO_4_ anode. Experimental data and theoretical calculation results indicate that the redox reaction of V^3+^/V^2+^ couple has remarkable priority over Nb^4+^/Nb^3+^ couple in the potential range of 1.0–1.5 V due to the stronger chemical affinity between Li and V atoms. Therefore, V^3+^/V^2+^ redox couple can effectively inhibit the irreversible over-reduction of Nb^5+^ to Nb^3+^, thus avoiding the rapid capacity decay in the long-term cycles. In addition, the electron migration from V^3+^ to Nb^5+^ leads to a superior intrinsic electronic conductivity for VNbO_4_. The significant enhancement of electronic conductivity and the reversibility of the redox reaction during charge/discharge processes endows VNbO_4_ electrode with fast and durable Li storage performances.

## Experimental and Methods

### Materials Synthesis

Firstly, 0.001 mol niobium chloride (NbCl_5_, 99.9%, Macklin) and 0.001 mol vanadium (III) 2,4-pentanedionate (C_15_H_21_O_6_V, 99.9%, Macklin) were added to the mixed solution of 35 mL ethanol and 5 mL deionized water to form a suspension. Then, added 0.6 g urea (CH_4_N_2_O, 99.9%, Macklin), 0.63 g oxalic acid (C_2_H_2_O_4_, 99.5%, Macklin) and 0.063 g F127 (99.9%, Sigma-Aldrich) under vigorous stirring, kept stirring at room temperature for 30 min until the solution became clear. The obtained green transparent solution was transferred to a 100 mL Teflon autoclave and reacted at 180 °C for 18 h. After cooling to room temperature, the mixture was filtered and washed with ethanol (C_2_H_5_OH, 99.9%, Damao chemical reagent factory) for 5 times, and put into a vacuum drying oven for overnight drying. After that, the obtained precursor was transferred to corundum crucible, kept at 600 °C for 3 h in Ar protected tubular furnace, and then cooled naturally to obtain black VNbO_4_ powder. For comparison, T-Nb_2_O_5_ was also prepared via same synthesis process without V source.

### Materials Characterizations

The phase and structure of the samples were analyzed by X-ray diffraction analyzer (XRD, Bruker D8 advanced, Cu, K) *α*, *λ* = 1.5418 Å), the finer crystal structure parameters are obtained by Rietveld refinement method. The micro-morphology of the samples was obtained by cold field emission electron microscope (SEM, Hitachi SU8010). The internal structure, lattice arrangement and element distribution of the material were detected by field emission transmission electron microscope (TEM, JEOL, 3200Fs, accelerating voltage = 300 kV). The valence states of the elements in the samples were obtained by X-ray photoelectron spectroscopy (XPS, Thermo ESCALAB 250XI. The electronic conductivity was collected by the four-probe powder resistivity tester (ST2722-sd). UV–*vis* spectra were obtained by PE lambda 750. BET specific surfaces were collected by JW-BK200C surface area and pore size analyzer (Beijing JWGB Sci. & Tech. Co., Ltd) at the liquid-nitrogen boiling point (77 K).

*In situ* XRD testing is based on a self-made in situ testing system. The LIB-XRD *in situ* cell module (Beijing Scistar Technology Co., Ltd.) was assembled by anode of carbon paper loaded with active substances and cathode of Li metal foil; while, Be foil is installed on the top of the in situ battery as a window for X-ray. In the initial charge discharge test, XRD diffraction spectra were continuously collected 2θ (degree) range of 10°–55° with step increment of 0.02° at a scanning rate of 0.06° s^−1^.

### Electrochemical Measurements

The electrode preparation process is as follows: Active materials, acetylene black (Guangdong Canrd New Energy Technology Co., Ltd.) and polytetrafluoroethylene (PVDF, Mw = 400,000, Macklin) were mixed with the ratio of 8:1:1. The slurry was obtained after added drops *N*-methyl-2-pyrrolidone (NMP, 99.5%, Macklin) and then coated on one side of copper foil. After drying for 12 h in 80 °C, the copper foil was punched as disc with a diameter of 13 mm. The mass of materials loading on each disc was about 1.3–1.4 mg. The CR2032 coin type cells were assembled in a glovebox and each cell was composed of the above electrode, separator (polyethylene membrane, Celgard), counter electrode (lithium tablet, China Energy Lithium Co., Ltd.) and electrolyte (1.0 M LiPF_6_ in EC: DMC: EMC = 1:1:1 vol%, www.dodochem.com). The LAND2001CT battery testing system was employed for galvanostatic charging/discharging measurements. And a constant temperature drying oven at 60 °C and a constant temperature refrigerator at − 10 °C were used as battery test boxes for electrochemical test in a wide temperature range.

The assembly process of full cell is the same as above, while replaced lithium foil with commercial cathode (LiFePO_4_@C: acetylene black: PVDF = 8:1:1) as counter electrode.

The assembly process of lithium-ion capacitor is the same as above, while replaced lithium foil with active carbon electrode (active carbon: acetylene black: PVDF = 8:1:1) as counter electrode.

The cyclic voltammetry (CV) test at a voltage range of 1.0–3.0 V and EIS test with a frequency range of 100,000–0.01 HZ was carried on a electrochemical workstation (CHI660E, Chenhua, Shanghai).

The energy/power density of lithium-ion capacitor can be obtained by Eqs. (1) and (2):

1 $$\mathrm{E }= \underset{t1}{\overset{t2}{\int }}\mathrm{IV}\left(t\right)\mathrm{dt}$$

2 $$\mathrm{P }= \frac{E}{t}$$

where *I* (A g^−1^) is the constant current density based on the total active materials, *V* (V) is the working voltage, *t1* and *t2* is the discharge start/end time (s), and *t* (s) is the discharging time of lithium-ion capacitor. *E* is the energy density and *P* is the power density.

### Density Functional Theory Calculation Method

All the calculations for structure optimization have been carried out with the Vienna Ab Initio Simulation Package (VASP) at the density functional theory (DFT) based with 3D periodic conditions. The exchange–correlation functional was treated by generalized gradient approximation (GGA) with the Perdew–Burke–Ernzerhof (PBE). The electron–ion interaction was treated by the projector-augmented wave method (PAW) that the core electrons were treated with cost-effective pseudopotentials implemented in VASP, and the valence electrons were expanded by plane-wave basis with the kinetic cutoff energy of 520 eV. The electronic energy was considered self-consistent when the energy change was smaller than 10^–5^ eV with a 4 × 1 × 3 Monkhorst–Pack k-point grid for Brillouin zone sampling. Grimme’s DFT-D3 methodology was used to describe the dispersion interactions. In addition, since the number and species of each atom are the same, we judge the trend based on the total energy of the model of the four sites where Li ions are inserted.

## Results and Discussion

### Physical Characterization Results

**Fig. 1:**
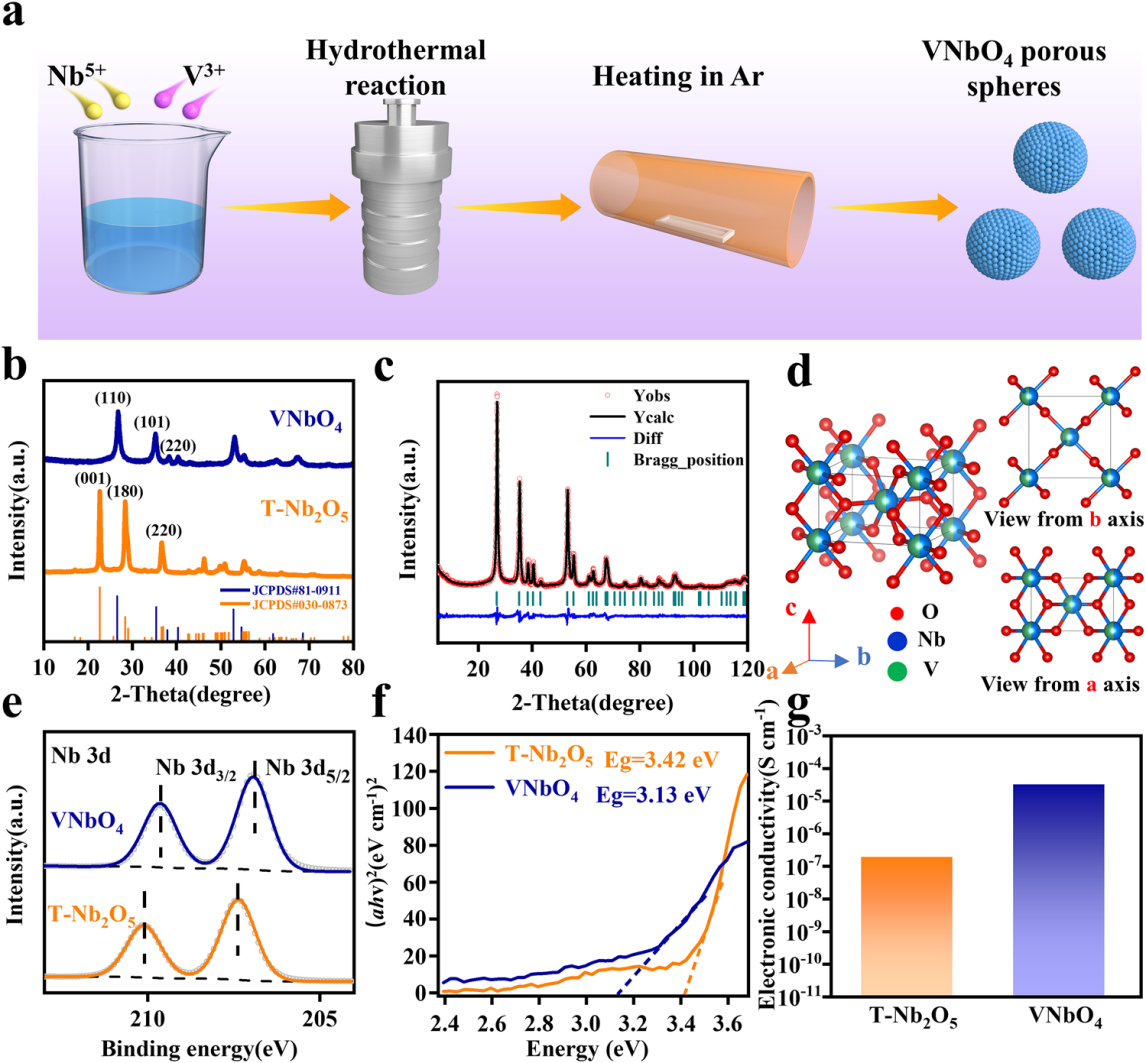
**a** Synthesis strategy of VNbO_4_. **b** XRD patterns of T-Nb_2_O_5_ and VNbO_4_. **c** Rietveld refinement pattern of VNbO_4_. **d** Crystal model of VNbO_4_. **e** The high-resolution Nb 3*d* XPS spectra of T-Nb_2_O_5_ and VNbO_4_. **f** Ultraviolet–visible spectroscopy and **g** the electronic conductivities of T-Nb_2_O_5_ and VNbO_4_ measured by four-probe method

The VNbO_4_ was prepared by a facile surfactant-assisted hydrothermal treatment of the mixed solution of NbCl_5_ and C_15_H_21_O_6_V with stoichiometric ratio followed by an annealing process in Ar flow, as shown in Fig. 1a. For comparison, T-Nb_2_O_5_ was also prepared via the same synthesis process without V source. The XRD spectra in Fig. 1b show the structural information of the as-prepared VNbO_4_ and T-Nb_2_O_5_. The diffraction peaks of VNbO_4_ at 26.9°, 35.3° and 39.2° correspond to (110), (101) and (220) planes, while the diffraction peaks of T-Nb_2_O_5_ at 22.5°, 28.5° and 36.7° correspond to (001), (180) and (200) planes, both of them being consistent with the previous reports [9, 23]. To understand the structure advantages of VNbO_4_ as electrode material, Rietveld refinement was employed to analyze the XRD pattern of VNbO_4_, as shown in Fig. 1c (Rietveld refinement pattern of T-Nb_2_O_5_ is exhibited in Fig. S1). The results show that VNbO_4_ belongs to rutile structure with *P42/mnm* space group (*a*, *b* = 4.6956 Å, *c* = 3.0336 Å, *α* = *β* = *γ* = 90°). The corresponding crystal model in Fig. 1d clearly shows that the open tunnel in the *c*-axis direction provides the efficient Li^+^ insertion site and transport channel.

Except for different crystal structure, the core-level Nb 3*d* XPS spectra reveal the different Nb valence states in T-Nb_2_O_5_ and VNbO_4_. As shown in Figs. S2 and 1e, the typical double peaks of Nb 3*d*_3/2_ and Nb 3*d*_5/2_ formed by the spin–orbit splitting are located at 210.1 and 207.4 eV in T-Nb_2_O_5_, which indicates that Nb element is existed in the highest oxidation state (Nb^5+^) [24]. In the VNbO_4_, Nb 3*d*_3/2_ and Nb 3*d*_5/2_ peaks are located at obviously lower binding energies of 209.6 and 206.8 eV, respectively, which demonstrates that the valence-shell electron state of Nb atoms in VNbO_4_ is different from that in T-Nb_2_O_5_. To further reveal the changed electron clouds of metal elements in VNbO_4_, the V 2*p* XPS spectrum is shown in Fig. S3. Two characteristic peaks of V^3+^ are located at 524.1 and 517.2 eV, respectively, which are higher than the typical binding energies in the previous reports [25, 26]. The abnormal binding energies of Nb 3*d* and V 2*p* XPS spectra can be attributed to the valence-shell electron migration from V^3+^ to Nb^5+^, in which V^3+^ serves as an electron donor due to its reduction ability and Nb^5+^ as an electron acceptor due to its oxidation ability. UV–vis tests were carried out to verify the influence of electron clouds transfer from V to Nb on the electronic conductivity. As displayed in Fig. 1f, the band gap of VNbO_4_ (3.13 eV) is obviously small than that of T-Nb_2_O_5_ (3.42 eV). Furthermore, four-probe method electronic conductivity tests were performed to obtain the detailed electronic conductivity. As shown in Fig. 1g, VNbO_4_ exhibits a high electronic conductivity of 3.11 × 10^−5^ S cm^−1^, two orders higher than that of T-Nb_2_O_5_ (1.85 × 10^−7^ S cm^−1^). Therefore, the spontaneous regulation of valence-shell electrons endows VNbO_4_ with greatly enhanced electronic conductivity.

### Morphological Characterization Results

**Fig. 2:**
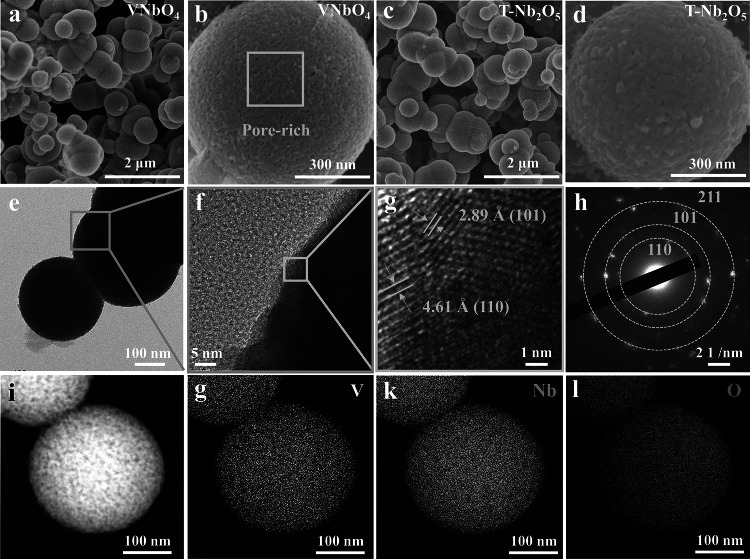
SEM images of **a**, **b** VNbO_4_ and **c**, **d** T-Nb_2_O_5_ at different magnifications. **e**, **f** TEM images, **g** HRTEM image and **h** SADE pattern of VNbO_4_. **i**–**l** EDS elements mapping images of VNbO_4_

The microstructures of VNbO_4_ and T-Nb_2_O_5_ were investigated via SEM and TEM. As shown in Fig. 2a–d, both of two samples show spherical morphology with a diameter of 300–700 nm as well as uniformly distributed pores. Brunauer–Emmett–Teller (BET) method was employed to obtain detailed information on the pore structure of T-Nb_2_O_5_ and VNbO_4_ (Fig. S2). The results show that both T-Nb_2_O_5_ and VNbO_4_ have mesoporous structure with the pore volume of about 0.06 m^3^ g^−1^ and the pore size of 3–20 nm. The BET results and TEM images in Figs. 2e and S4 illustrate that the pores on the surface are extended to the bulk, which can facilitate the full infiltration of electrolyte and provide buffer space for volume expansion of during the charge process [27]. High resolution TEM images show both T-Nb_2_O_5_ and VNbO_4_ have high crystallinity (Figs. 2f–g and S5). The VNbO_4_ exhibits two kinds of crystal plane spacing of 4.61 and 2.89 Å, which correspond to (110) and (101) planes of VNbO_4_, respectively. The selected-area electron diffraction (SAED) pattern exhibited in Fig. 2h demonstrates that VNbO_4_ possesses a polycrystalline structure with well-defined diffraction rings of (110), (101) and (211) planes. The element distribution of VNbO_4_ can be characterized by the energy dispersive spectrometer (EDS). As shown in Fig. 2i–l, V, Nb and O elements are very evenly distributed in the whole spherical particle.

### Electrochemical Properties in Half-Cell Tests

**Fig. 3:**
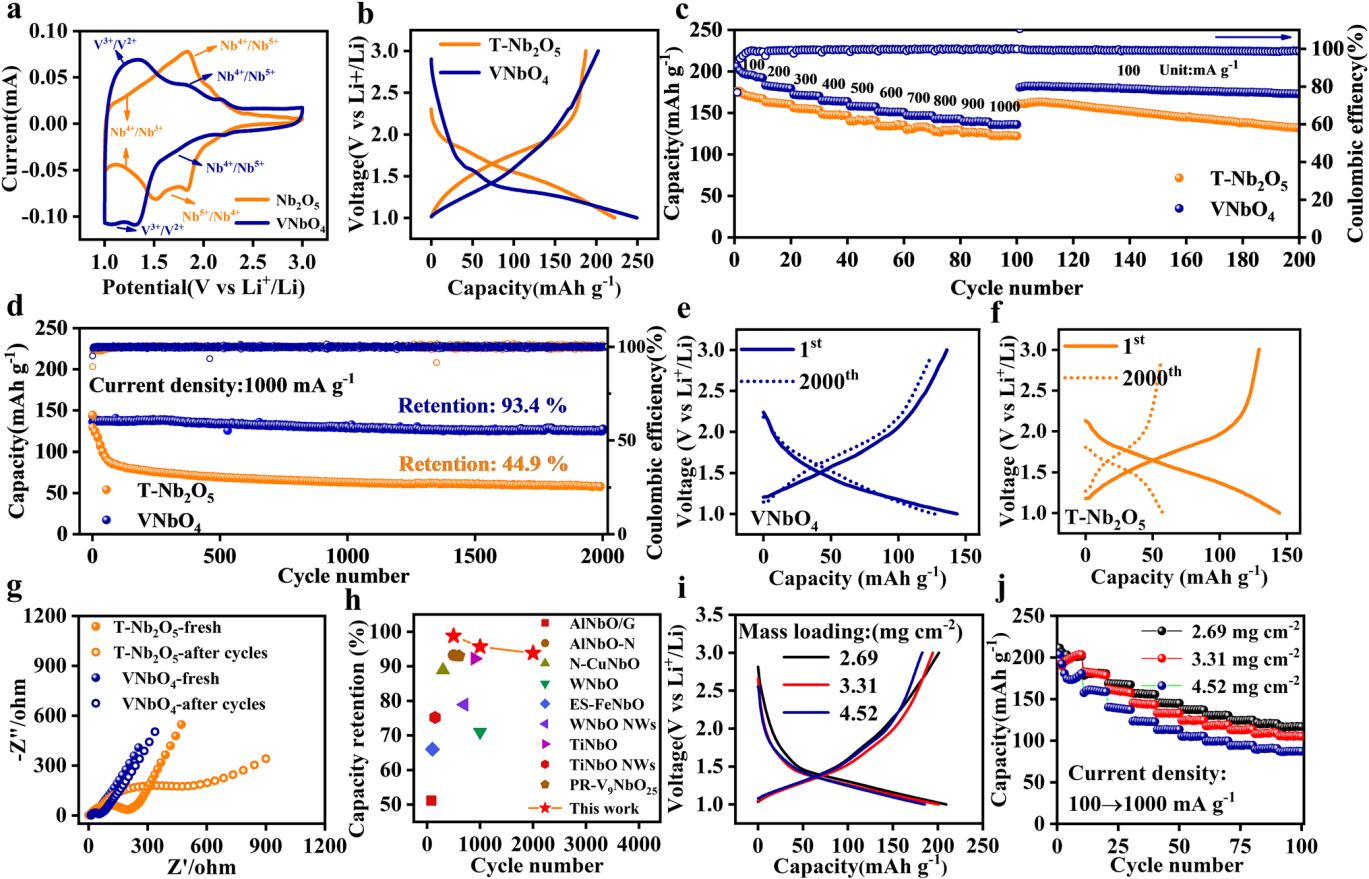
**a** The initial CV curves at a sweep rate of 0.1 mV s^−1^, **b** the initial charge–discharge profiles at 0.1 A g^−1^, **c** the rate performances and the subsequent cycle performance at 100 mA g^−1^, **d** the cycle performance at 1.0 A g^−1^ of VNbO_4_ and T-Nb_2_O_5_ anodes. The selected charge–discharge profiles at 1.0 A g^−1^ of **e** VNbO_4_ and **f** T-Nb_2_O_5_. **g** The AC impedance of two electrodes before and after 2000 cycles. **h** The comparison of cycle retention between VNbO_4_ with previous works. **i** The charge–discharge profiles at 0.1 A g^−1^ and **j** the rate capacities of VNbO_4_ with different mass loading

Then, the Li storage performances of VNbO_4_ were evaluated in half cell in the potential range of 1.0–3.0 V and made a comparison with those of T-Nb_2_O_5_. The Li storage mechanism of the VNbO_4_ was firstly investigated by *in situ* XRD on a live, specially designed cell with VNbO_4_ anode working at a current density of 0.1 A g^−1^. As shown in Fig. S6, when the cell was discharged from open circuit voltage (OCV) to 1.0 V, the characteristic peaks of (110), (200), (211), (002) and (301) shift to the lower degrees, which is caused by the Li^+^ insertion into host VNbO_4_, as observed in other insertion-type anodes [28, 29]. During the following charge process from 1.0 to 3.0 V, a full positive shift can be observed for the main diffraction peaks of VNbO_4_. Similarly with Nb_2_O_5_, VNbO_4_ also possesses the reversible solid-solution-like Li storage behavior without phase transformation, which can be confirmed by the *ex situ* HRTEM images of VNbO_4_ anode at different discharge/charge states (Fig. S7). However, VNbO_4_ demonstrates the different redox chemistries from Nb_2_O_5_, as illustrated by the CV curves in Fig. 3a. Similar with the typical pseudocapacitive RuO_2_ anode [30–32], T-Nb_2_O_5_ demonstrates the typical broad redox peaks in potential range of 1.3–2.1 V, which can be attributed to the stepwise redox process from Nb^5+^ to Nb^4+^ and then Nb^3+^ cause by the Li^+^ insertion [33, 34]. However, the most obvious redox peaks for VNbO_4_ are located at a low potential range of 1.0–1.5 V, which corresponds to the redox couple of V^3+^/V^2+^, The peak area contributed by Nb redox reaction in potential range of 1.3–2.1 V is significantly reduced, which indicates that the capacity contribution from the redox reaction of Nb is reduced [35]. Besides, the CV curves at different sweep rates shown in Fig. S8 demonstrate that VNbO_4_ still shows intercalation pseudocapacitive charge-storage behavior. Meanwhile, the initial charge–discharge curves at 0.1 A g^−1^ show that VNbO_4_ and T-Nb_2_O_5_ exhibit similar initial Coulombic efficiency of about 80% (Fig. 3b). The large capacity loss may be attributed to the Li^+^ inserted the disordered defect area in the crystal, as well as the conductive carbon in the electrode [36, 37]. Specifically, VNbO_4_ anode can deliver a high charge capacity 206.1 mAh g^−1^, which is higher than T-Nb_2_O_5_ anode (179.1 mAh g^−1^). Meanwhile, VNbO_4_ anode shows a lower insertion/extraction Li plateau than T-Nb_2_O_5_, which agrees well with CV curve.

Besides, VNbO_4_ also delivers improved rate performances with slight increase in potential polarization at various current densities, as displayed in Figs. 3c and S9. For example, VNbO_4_ remains a remarkable capacity of 136.2 mAh g^−1^ at 1.0 A g^−1^, while T-Nb_2_O_5_ only shows a capacity of 123.3 mAh g^−1^. Noting that VNbO_4_ can maintain a high retention ratio of 96.0% after 100 cycles when the current density was reset to 0.1 A g^−1^, much higher than that of T-Nb_2_O_5_. The excellent cycle stability for VNbO_4_ is also verified by the cycle performance at 1.0 A g^−1^. As exhibited in Fig. 3d–e, VNbO_4_ demonstrates an impressive capacity retention of 93.4% after 2000 cycles at high current density of 1.0 A g^−1^, and the charge–discharge curve at 2000th cycle is well maintained. In sharp contrast, T-N_2_O_5_ experiences rapid capacity deterioration in the initial tens of cycles and a poor capacity retention of 44.9% is obtained after 2000 cycles with greatly increased polarization (Fig. 3f). Compared to the previous reported Nb-based bimetallic transition oxides (AlNbO_4_/G [38], AlNb_11_O_29_-N [39], N-Cu_2_Nb_34_O_87_ [40], WNb_12_O_33_ [24], ES-FeNbO_4_ [41], WNb_12_O_33_ NWs [42], TiNb_24_O_62_ [43], TiNb_6_O_17_ NWs [44], PR-V_9_NbO_25_ [45]), the performance of VNbO_4_ anode designed in this study stands among the best in cycle stability, which can be also proved by the excellent electrochemical performances under high mass loading (Figs. 3g and S10) as well as at extreme temperatures (Fig. S11). At a loading of 4.52 mg cm^−2^, a remarkable reversible capacity of 203.7 mAh g^−1^ is delivered at 0.1 A g^−1^ without obvious increased overpotential (Fig. 3h). Even at an elevated current density of 1.0 A g^−1^, a capacity of 87.3 mAh g^−1^ can be still delivered (Fig. 3i).

To understand the different cycle stability between VNbO_4_ and T-Nb_2_O_5_, the surface morphologies of two electrodes at different cycles were firstly characterized (Fig. S12). The SEM images of cycled VNbO_4_ and T-N_2_O_5_ show that their sizes increase slightly without obvious pulverization after 500 and 2000 cycles, indicating that both of them have excellent structural stabilities during the repeated Li^+^ insertion/extraction processes. Then the electrochemical impedance spectra (EIS) were conducted on electrodes with different cycle numbers (Fig. 3j). The charge transfer impedance (*R*_ct_) of VNbO_4_ electrode at initial state is obviously lower than T-N_2_O_5_ electrode, and experiences a slight increase after 2000 cycles (Fig. S13). In vast contrast, the *R*_ct_ of T-N_2_O_5_ electrode at 2000th cycle is significantly increased compared to the initial cycle. These results indicate that VNbO_4_ can maintain good electron transfer in the whole electrochemical cycles.

### Mechanism Discussion

**Fig. 4:**
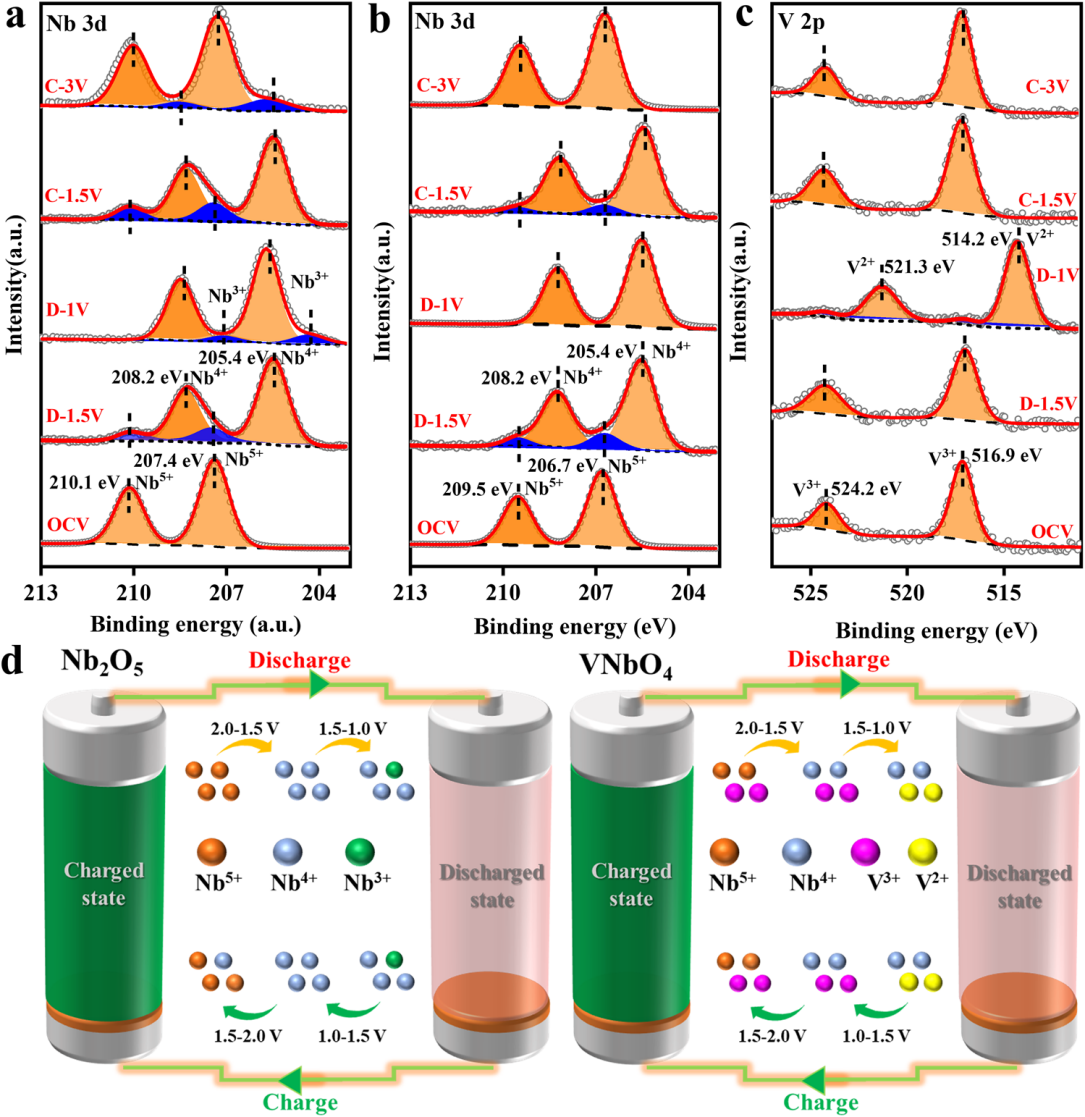
*Ex situ* XPS spectra of **a** Nb 3*d* in T-Nb_2_O_5_, **b** Nb 3*d* and **c** V 2*p* in VNbO_4_ anode at different lithiated/delithiated states. **d** Schematic diagram of redox chemistries in different anodes

Furthermore, e*ex situ* XPS was also carried out to analyze the valences of cation in both T-Nb_2_O_5_ and VNbO_4_ at different lithiated/delithiated states in the initial charge–discharge process. For T-Nb_2_O_5_ at OCV state, the characteristic peaks of Nb 3*d* are located at 210.1 and 207.4 eV (Fig. 4a). After discharging to 1.5 V, the dominant peaks of Nb 3*d* shift to low binding energies of 208.2 and 205.4 eV, which represents that most Nb^5+^ is reduced to Nb^4+^. The existence of weak doublet related to Nb^5+^ implies that the reduction reaction from Nb^5+^ to Nb^4+^ is not be completed at 1.5 V, and the characteristic peaks of Nb^5+^ are disappeared when discharged to 1.0 V. Meanwhile, a small doublet of Nb^3+^ can be observed at 207.4 and 204.9 eV, which suggests that partial Nb^4+^ is further reduced in the low potential range of 1.5–1.0 V. In the following delithiation process, the lithiation product Nb^4+^ is gradually converted into Nb^5+^. However, Nb^4+^ is still observed even after a constant-potential charging process at 3.0 V for 30 min. Considering the Nb^3+^ will experience a two-step oxidation process to Nb^5+^ in the following delithiation process, the existence of Nb^4+^ in the full charged T-Nb_2_O_5_ state is largely attributed to the formation of Nb^3+^ in the lithiation process. Besides, the Nb 3*d* orbit XPS spectra of T-Nb_2_O_5_ electrode at different lithiated/delithiated states in the 50th and 100th charge–discharge process at 1 A g^−1^ strongly proves that Nb^4+^/Nb^3+^redox couple are not as reversible as Nb^5+^/Nb^4+^ (Fig. S14). Therefore, the over-reduction of Nb^5+^ to Nb^3+^ should be main reason for the capacity loss for T-Nb_2_O_5_ anode. However, ex situ Nb 3*d* XPS spectra of VNbO_4_ at different lithiated/delithiated states show remarkable differences. As shown in Fig. 4b, no Nb^3+^ is occurred in the voltage window of 1.0–1.5 V in the lithiation process and the Nb^4+^ can be completely converted into Nb^5+^ in the following delithiation process. Meanwhile, V^3+^ will be reduced to V^2+^ is in the voltage range of 1.0–1.5 V (Fig. 4c). Obviously, the redox couple of V^3+^/V^2+^ has a higher priority over that of Nb^4+^/Nb^3+^ couple in the range of 1.0–1.5 V. To provide more intuitive proof, the models of NbO_2_ and V_2_O_3_ was created to calculate the energy barrier of Nb^4+^/Nb^3+^ and V^3+^/V^2+^, as shown in Fig. S15. Through calculating the energy difference between the models before and after electron injection, it can be concluded that the energy barrier for V^3+^ to V^2+^ is 1.07 eV, which is significantly lower than the energy barrier for Nb^4+^ to Nb^3+^(2.39 eV). Therefore, the reduction of V^3+^ in VNbO_4_ takes priority over Nb^4+^ and can effectively suppress the generation of Nb^3+^ during the long-term cycling processes. Figure 4d shows the different redox chemistries in T-Nb_2_O_5_ and VNbO_4_ during the electrochemical process. In the discharge process, some Nb^5+^ in T-Nb_2_O_5_ electrode will be over reduced to Nb^3+^, and Nb will not return to the initial valence state in the following charge process. However, the introduction of V^3+^/V^2+^ couple can effectively inhibit the over-reduction of Nb^5+^. As a result, both Nb and V in VNbO_4_ electrode exhibit highly reversible redox chemistries in the charge–discharge processes.

**Fig. 5:**
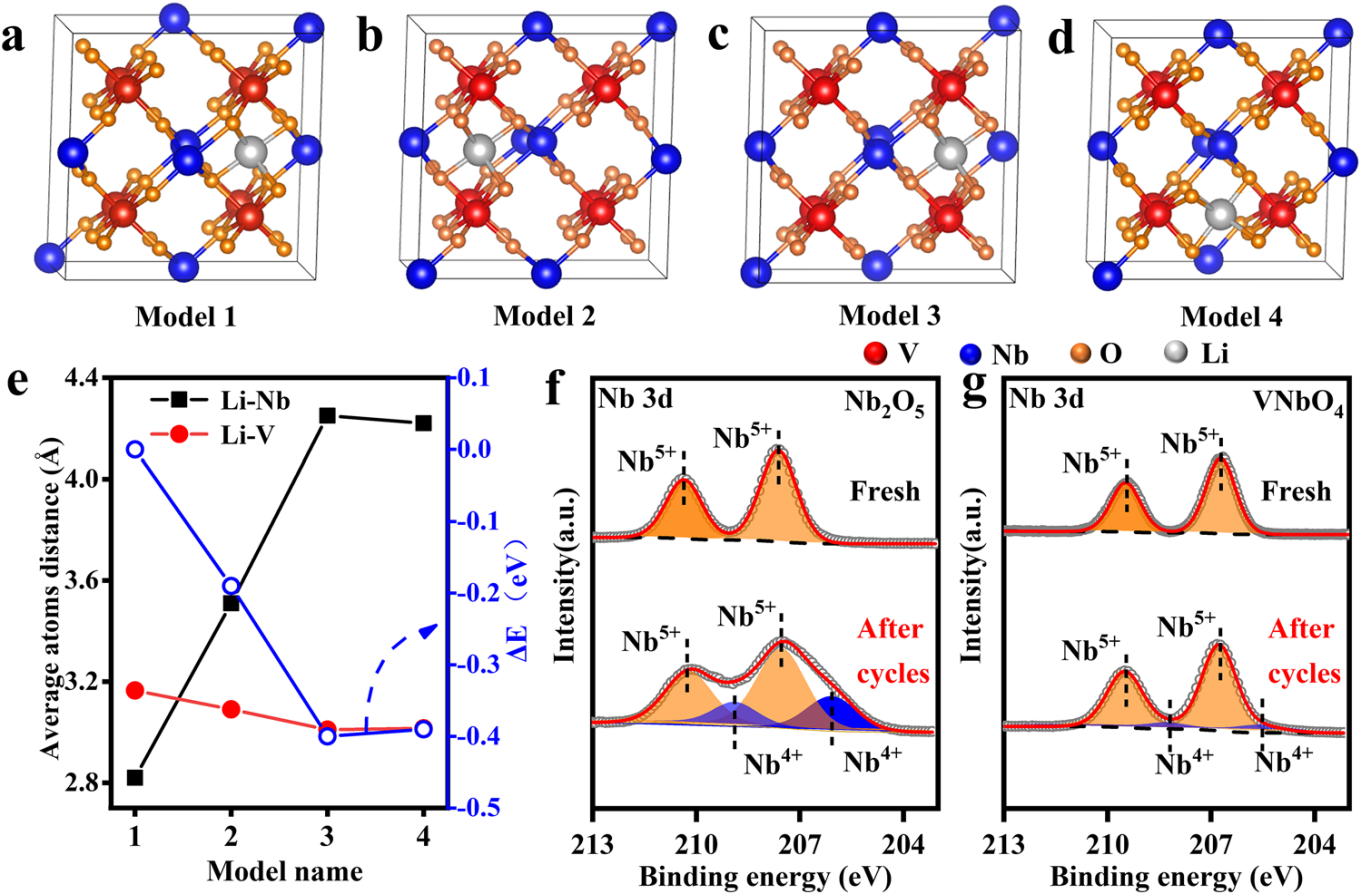
**a**–**d** Crystal models of VNbO_4_ with different lithium-insert sites. **e** Response of model energy to Li–Nb atomic distance or Li-V atomic distance. Nb 3*d* XPS spectra of **f** Nb_2_O_5_ and **g** VNbO_4_ anode after 1000 cycles

DFT calculation was employed to demonstrate the higher priority of V^3+^/V^2+^ couple in the charge/discharge process via evaluating the affinity of V and Nb atoms to Li atoms. Four models with different Li insertion sites were created and the distance between Li and adjacent V atoms in each model and corresponding overall energy were calculated (Fig. 5a-d). The atomic distances of Li and V in four models are 3.165, 3.09, 3.01 and 3.015 Å, respectively (Table S2). The overall energy of each model is normalized based on the energy of model 1. The energy differences (ΔE) between model 2, 3 and 4 and model 1 are -0.19, -0.4 and -0.39 eV, respectively. Notice that the average distance between Li and V atom in model 3 is minimal, and the overall energy is the lowest (Fig. 5e), indicating that Li atoms are more inclined to choose the insertion site closer to V atom. Therefore, the highly reversible redox behavior of VNbO_4_ can be attributed to the competitive redox behavior caused by the introduction of V^3+^/V^2+^ couple. In the potential range of 1.5 ~ 3.0 V, the redox conversion between Nb^5+^ and Nb^4+^ dominates the capacity contribution, while the redox reaction of V^3+^/V^2+^ couple take priority over Nb^4+^/Nb^3+^ couple in the potential range of 1.0 ~ 1.5 V owing to the stronger chemical affinity between Li atoms and V atoms. As results, VNbO_4_ anode delivers excellent cycle stability via suppressing the over-reduction of Nb^5+^.

To further demonstrate the conclusion, the post-mortem XPS analysis of VNbO_4_ and T-Nb_2_O_5_ electrodes at fully charged state after 1000 cycles was carried out. As shown in Fig. 5f, a considerable amount of Nb in T-Nb_2_O_5_ cannot return to the initial Nb^5+^ state after long-term discharge–charge cycles, indicating that the irreversible redox chemistry of Nb is the main reason for the capacity decay. In sharp contrast, Nb^5+^ in VNbO_4_ exhibits excellent reversibility after 1000 cycles (Fig. 5g). In addition, V also maintains a high reversibility in the long-term cycles (Fig. S16).

### Electrochemical Properties in Practical Energy Storage Devices

**Fig. 6:**
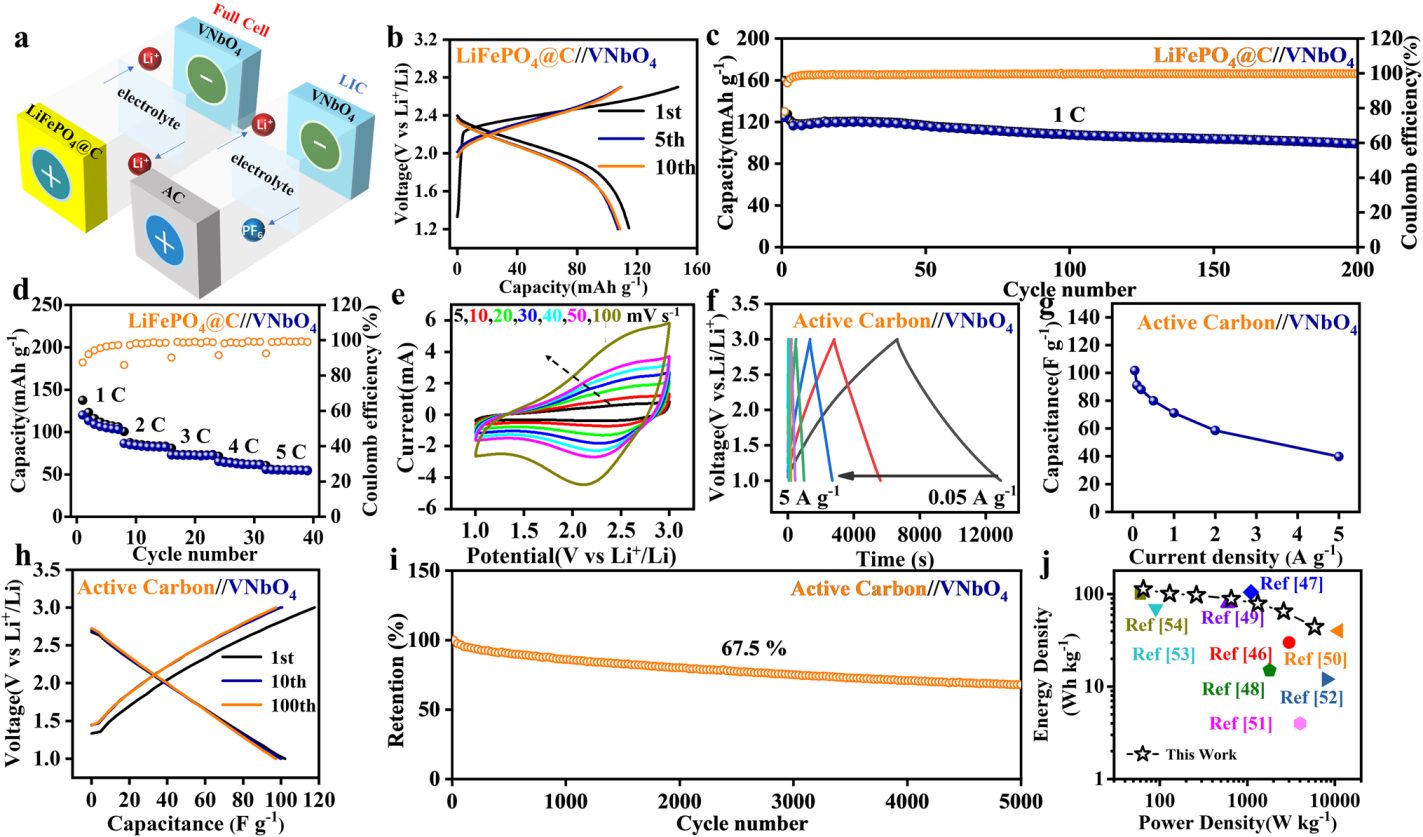
**a** Models diagram of LiFePO_4_@C//VNbO_4_ full cell and AC//VNbO_4_ LIC. **b** The selected charge/discharge profiles, **c** the cycle performance at 1C, **d** the rate performance of LiFePO_4_@C//VNbO_4_. **e** CV curves at different sweep rates (5–100 mV s^−1^) and **f** the time-potential curves at different current densities of AC//VNbO_4_. **g** Rate performance, **h** the selected charge/discharge profiles at 0.05 A g^−1^, and **i** the cycle performance at 0.5 A g^−1^ of AC//VNbO_4_. **j** The Ragone plot of AC//VNbO_4_ compared with other reported niobium-based LICs

Lastly, the potential of practical application for VNbO_4_ anode was evaluated by assembling a full cell with commercial LiFePO_4_@C cathode and a lithium-ion capacitor (LIC) with active carbon (AC) cathode, respectively (Fig. 6a). The electrochemical performances of LiFePO_4_@C and AC in half cell (Li foil as counter electrodes) are shown in Figs. S17 and S18.

The LiFePO_4_@C//VNbO_4_ full cell can deliver a discharge capacity of 122.6 mAh g^−1^ at 1C (1C = 170 mA g^−1^) with an initial coulomb efficiency of 77.1% in potential range from 1.2 to 2.75 V (Fig. 6b). After 200 cycles at 1C, LiFePO_4_@C//VNbO_4_ remains a high discharge capacity of 97.8 mAh g^−1^ with a high retention of 79.8% (Fig. 6c). In a sharp contrast, LiFePO_4_@C//T-Nb_2_O_5_ delivers a poor capacity of 45.5 mAh g^−1^ with retention of 38.5% after 190 cycles at 1C (Fig. S19). The full cell also demonstrates a superior rate performance with high capacities of 98.6, 83.1, 74.4, 63.7 mAh g^−1^ at 2C, 3C, 4C and 5C, respectively (Fig. 6d). The electrochemical performances of VNbO_4_ anode in LIC were also studied in the potential range of 1.0–3.0 V. The overall mass loading of the two electrodes is about 4.5 mg cm^−2^. Figure 6e shows the asymmetric CV curves of AC//VNbO_4_ at different sweep rates of 5–100 mV s^−1^, which exhibit a quasi-rectangular shape, illustrating that the fast physical adsorption/desorption of PF_6_^−^ at the AC side and the relative sluggish intercalation/deintercalation kinetic of Li^+^ at the VNbO_4_ side are combined. In the following rate performance analysis (Fig. 6f-g), the LIC device can exhibit high capacitances of 103.3, 90.4, 87.9, 80.6, 72.1, 59.5 and 39.9 F g^−1^ at 0.05, 0.1, 0.2, 0.5, 1.0, 2.0 and 5.0 A g^−1^ respectively. As exhibited in Fig. 6h, the selected charge/discharge plots are highly overlapped, demonstrating the excellent reversibility of electrochemical behaviors of AC//VNbO_4_. Thus, the LIC device constructed by AC cathode and VNbO_4_ anode can deliver superior cycle stability with a retention of 67.5% after 5000 cycles (Fig. 6i). Ragone plot was obtained to clarify the relation of energy density and power density (Fig. 6j). AC//VNbO_4_ can deliver a high energy density of 113 Wh kg^−1^ at 65 W kg^−1^ as well as an ultra-high power density of 5.8 kW kg^−1^ at 44 Wh kg^−1^, which are much higher than many LICs previously reported [46–54]. In general, VNbO_4_ shows excellent electrochemical performance in both LIB and LIC systems, which can be considered as a potential candidate of the next generation anode materials.

## Conclusions

In this work, the over-reduction of Nb^5+^ in the lithiation process and the incomplete oxidation in the following delithiation process has been demonstrated to be a critical reason for the capacity decay of T-Nb_2_O_5_ for the first time. Then a competitive redox strategy has been proposed to suppress the over-reduction of Nb^5+^, which has been achieved by the incorporation of vanadium to form a new rutile VNbO_4_ anode. Due to the stronger chemical affinity Li atoms and V atoms, the redox conversion of V^3+^/V^2+^ couple has a higher priority than that of Nb^4+^/Nb^3+^ in VNbO_4_ anode during the lithiation/delithiation process. Therefore, the competitive redox strategy can effectively inhibit the over-reduction of Nb^5+^ to Nb^3+^ in VNbO_4_, achieving a highly reversible redox chemistry in the long-term cycling processes. Besides, the spontaneous electron migration from V^3+^ to Nb^5+^ can greatly enhance the electronic conductivity VNbO_4_ anode. As results, VNbO_4_ electrode exhibits promising application potentials in both practical LIB and LIC devices, which indicates that competitive redox strategy provides a new idea for designing fast and durable anodes.

### Supplementary Information

Below is the link to the electronic supplementary material.

Supplementary file1 (PDF 1844 KB)
