# Supplementary material for: Competitive Redox Chemistries in Vanadium Niobium Oxide for Ultrafast and Durable Lithium Storage
Source: Nanomicro Lett. 2023 Aug 10;15:195. doi: 10.1007/s40820-023-01172-9 (PMC10415248; doi:10.1007/s40820-023-01172-9)
Supplement: Supplementary file 1 — Supplementary file1 (PDF 1844 KB) [file 40820_2023_1172_MOESM1_ESM.pdf]

Supporting Information for

Competitive Redox Chemistries in Vanadium Niobium Oxide for Ultrafast and Durable Lithium Storage

Xiaobo Ding[1], Jianhao Lin[1], Huiying Huang[1], Bote Zhao[1], Xunhui Xiong[1,]*

[1]School of Environment and Energy, Guangdong Provincial Key Laboratory of Advanced Energy Storage Materials, South China University of Technology, Guangzhou, 510640, P. R. China

*Corresponding author. E-mail: esxxiong@scut.edu.cn (Xunhui Xiong)

Supplementary Figures and Tables

Table S1 Cycle performances of Nb_2O_5 anodes in other reports

Cycle test at low current denstiy					Cycle test at high current denstiy				
Name	Cycle number	Current density (A g^{-1})	Initial capacity (mAh g^{-1})	Retention	Cycle number	Current density (A g^{-1})	Initial capacity (mAh g^{-1})	Retention	Reference
H-Nb_2O_5	100	0.2	235	74.6%	/	/	/	/	[S1]
3DOM Nb_2O_5	/	/	/	/	100	2	138	90.0%	[S2]
T-Nb_2O_5@C	1000	0.1	/	82.2%	/	/	/	/	[S3]
T-Nb_2O_5@CC	1000	1		86.0%	1000	2	/	85.0%	[S4]
Nb_2O_5 belt	50	0.1	200	88.5%	200	1	125	76.6%	[S5]
Wired H-Nb_2O_5	200	0.1	160	75.0%	/	/	/	/	[S6]
Nb_2O_5-600	/	/	/	/	2000	1	/	85.0%	[S7]
T-Nb_2O_5/rGO	100	0.1	227	72.6%	/	/	/	/	[S8]
Nb_2O_5	/	/	/	/	100	1	/	85.0%	[S9]
Nb_2O_5@NbO_2	900	0.2		81.0%	/	/	/	/	[S10]
Nb_2O_5@C/rGO	300	0.1	221	85.6%	/	/	/	/	[S11]
Nb_2O_5 NPs	/	/	/	/	1000	1	/	88.0%	[S12]
YS-Nb_2O_5	200	0.1	205	85.30%	/	/	/	/	[S13]
N-NbOC	1100	0.5	/	81.0%	/	/	/	/	[S14]
Nb_2O_5/C	100	0.1	559.8	43.9%	300	/			[S15]
T-Nb_2O_5/CNTs	500	2	/	83.0%	500	10	/	80.0%	[S16]
NC-Nb_2O_5	/	/	/	/	1000	2	196	83.0%	[S17]
T-Nb_2O_5 NRs	270	0.1	257	62.3%	4000	0.5	226	40.7%	[S18]
M-Nb_2O_5	1000	0.2	160	82.3%	/	/	/	/	[S19]

Nb_2O_5@C	500	0.1	/	72.0%	/	/	/	/	[S20]
TT-Nb_2O_5	/	/	/	/	1000	1	103	80.7%	[S21]
Nb_2O_5-C-rGO	/	/	/	/	1500	1	192	76.6%	[S22]
NbW-3/MXene	/	/	/	/	500	1	/	59.8%	[S23]
H-Nb_2O_5	100	0.05	260	73.2%	/	/	/	/	[S24]
CN-NbO	550	0.1	340	79.4%		1	230	71.3%	[S25]
Nb_2O_5 NC	190	0.1	224	87.0%	2000	2	151	66.9%	[S26]
T-Nb_2O_5	100	0.2	184	63.5%	200	1	92	65.2%	[S27]
T-Nb_2O_5	50	0.05	200	47.5%	/	/	/	/	[S28]
T-Nb_2O_5@C	1000	1	110	74.5%	/	/	/	/	[S29]
T-Nb_2O_5/rGO	/	/	/	/	3000	5	200	80 %	[S30]
Nb_2O_{5-x}	200	0.2	203.4	94%	1000	2	159	74.8%	[S31]
Nb_2O_5-5mM	100	0.2	285	88.4%	/	/	/	/	[S32]
CNF/NbO	100	0.2	619.2	58.3	/	/	/	/	[S33]
Nb_2O_5	200	1	105	94%	200	2	70	92%	[S34]
N- Nb_2O_5	100	0.1	/	83%	1000	1	/	70.5%	[S35]
N-Nb_2O_5	/	/	/	/	1000	2	/	90.1%	[S36]
Nb_2O_5-750	200	0.2	310	87%	800	1	/	52%	[S37]
Nb_2O_5@NC	/	/	/	/	2900	2	/	43%	[S38]

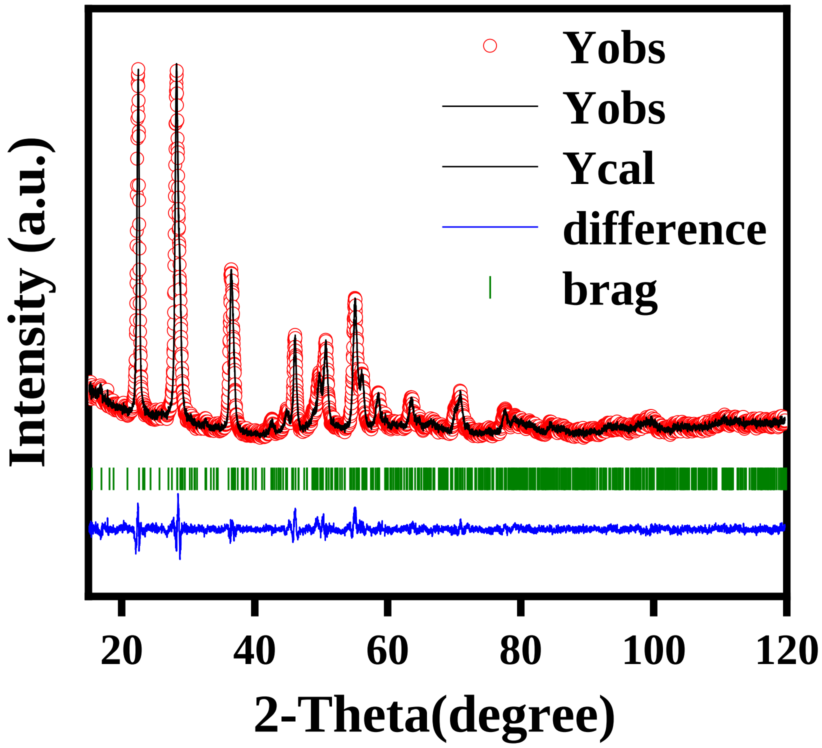

Fig. S1 Rietveld refinement pattern of T-Nb_2O_5

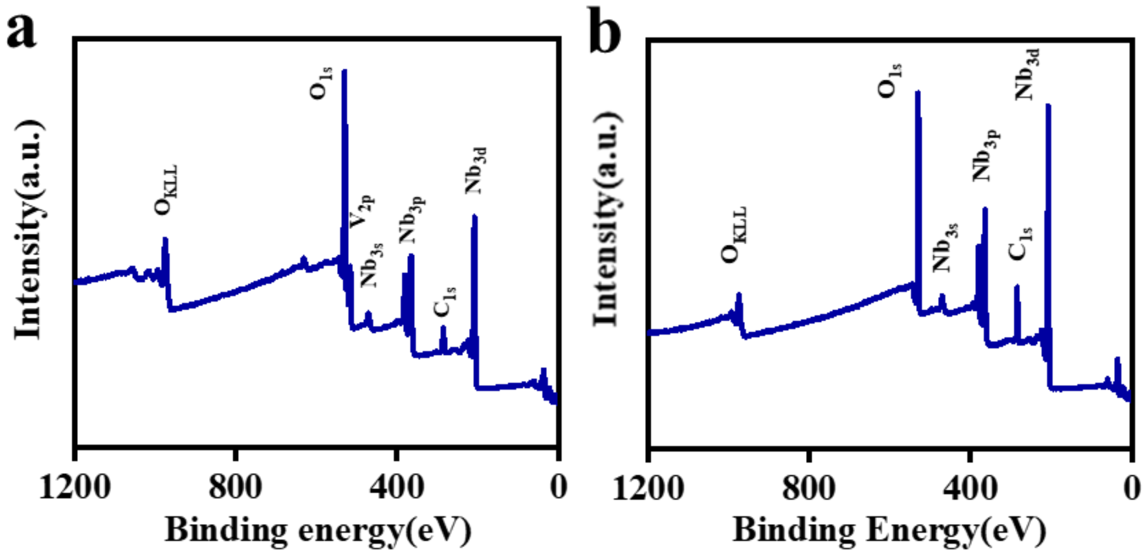

Fig. S2 XPS spectra of **a** $VNbO_4$. **b** T-Nb_2O_5

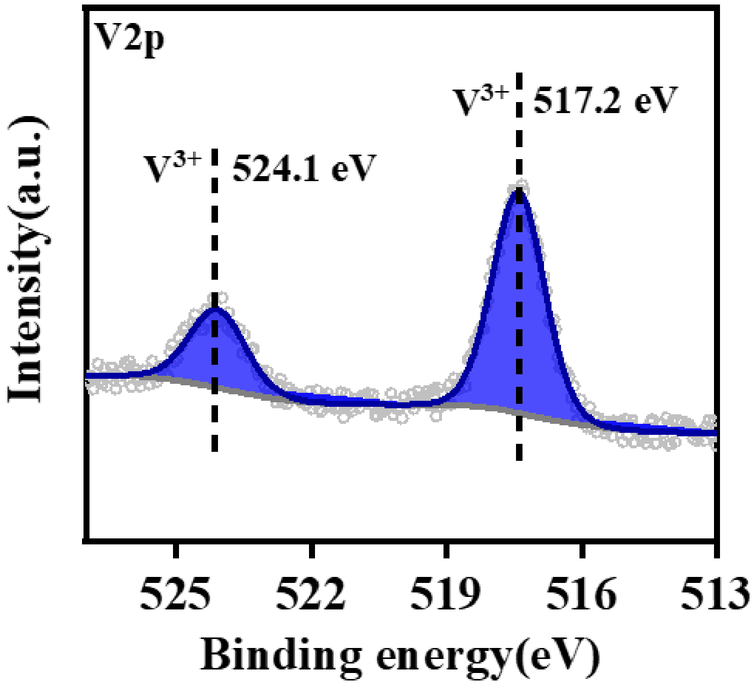

Fig. S3 Core-level V 2p XPS spectra of $VNbO_4$

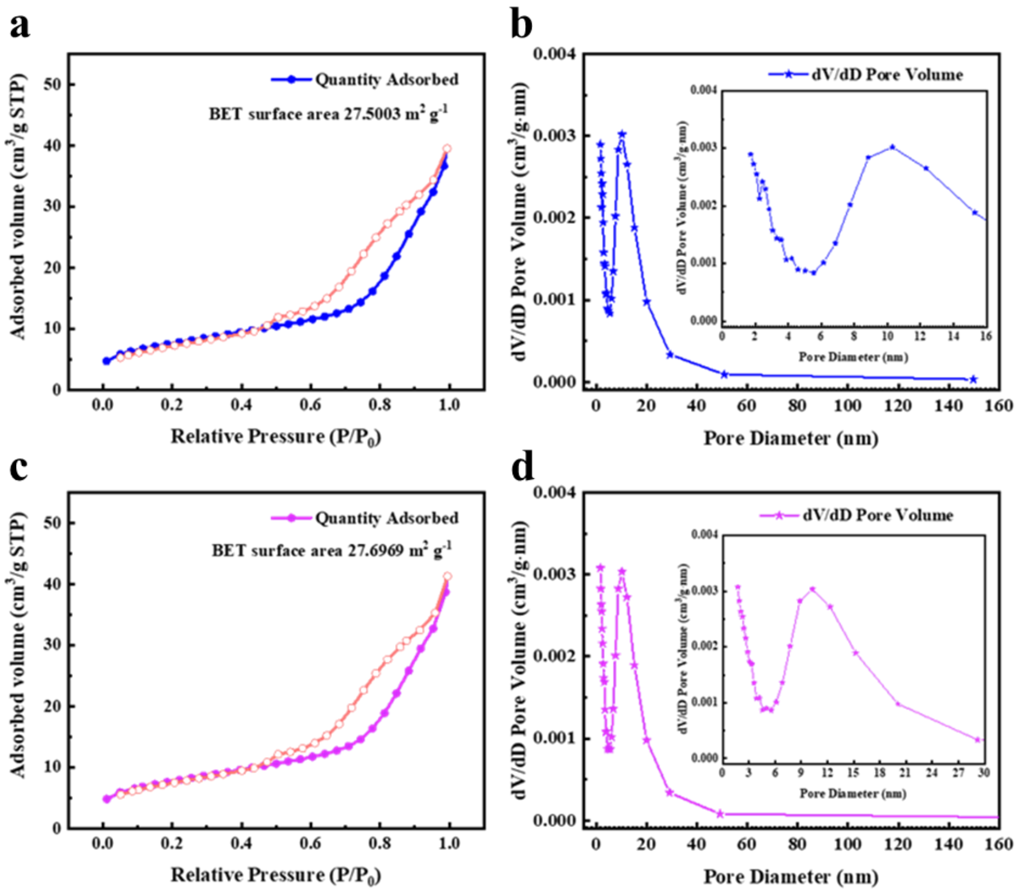

Fig. S4 N_2 adsorption–desorption isotherms of and the pore size distribution. **a** and **b** T-Nb_2O_5, **c** and **d** $VNbO_4$

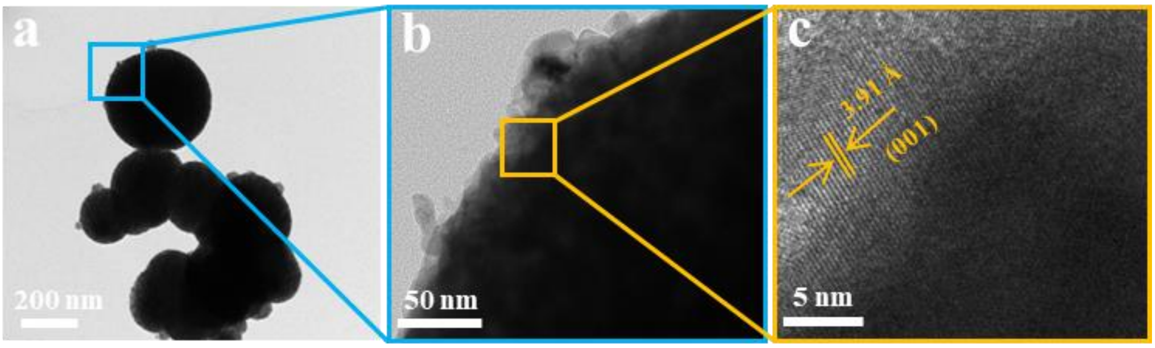

Fig. S5 **a** and **b** TEM images of T-Nb_2O_5. **c** HRTEM image of T-Nb_2O_5

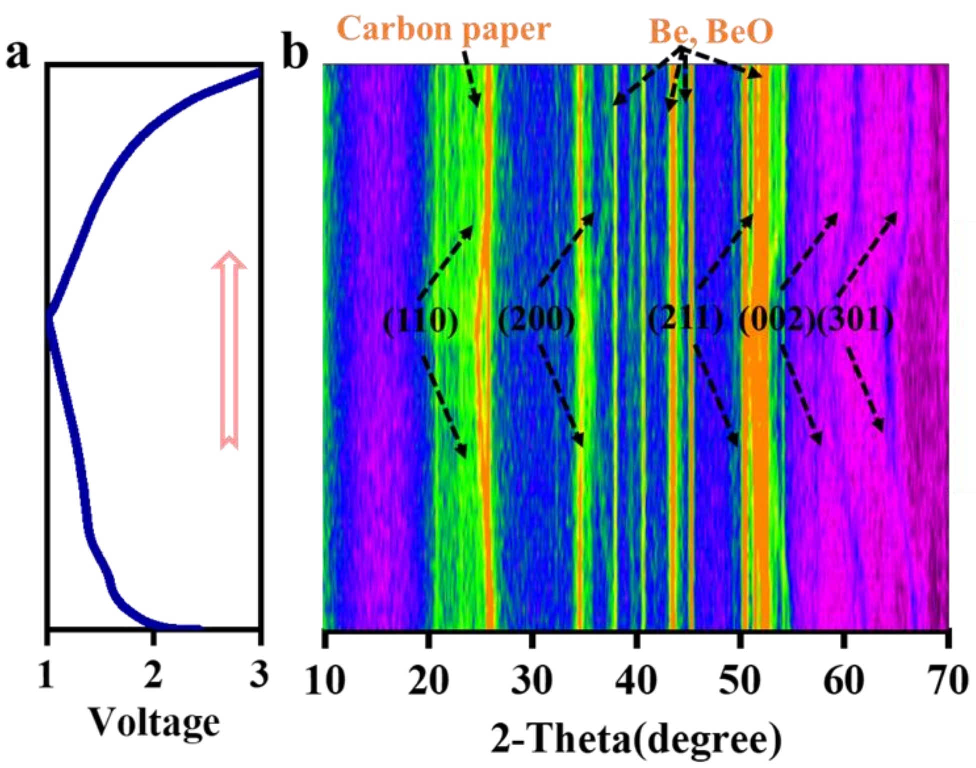

Fig. S6 Lithium storage mechanism and structure evolution of $VNbO_4$. **a** Electrochemistry profile corresponding to *in-situ* XRD test. **b** The 2D contour of *in-situ* XRD pattern at different lithiation/delithiation states of initial cycle in potential range of 1.0-3.0 V

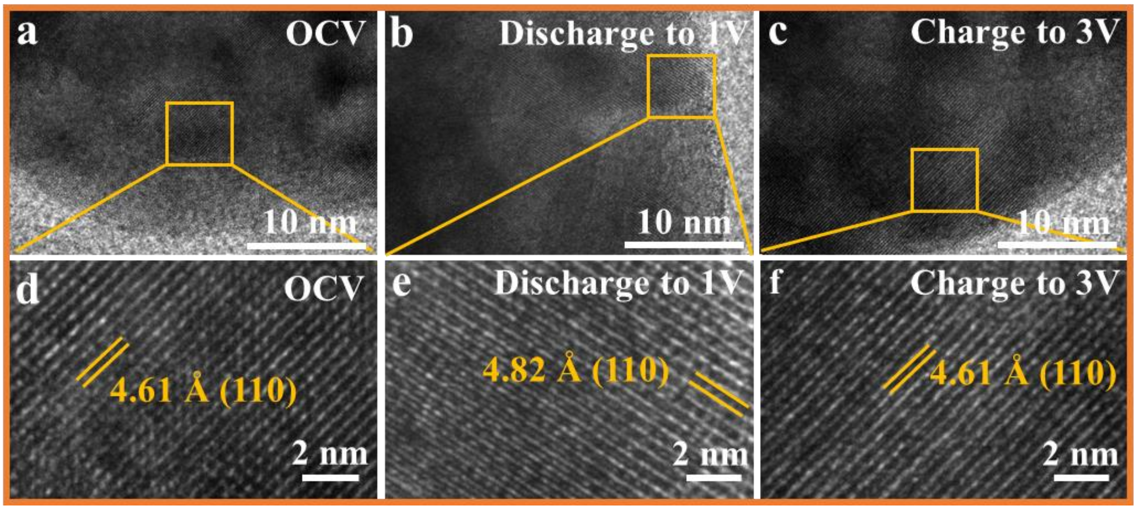

Fig. S7 a-c *Ex-situ* TEM images of $VNbO_4$ at different lithiation/delithiation states. **d-f** *Ex-situ* HRTEM images of $VNbO_4$ at different lithiation/delithiation states

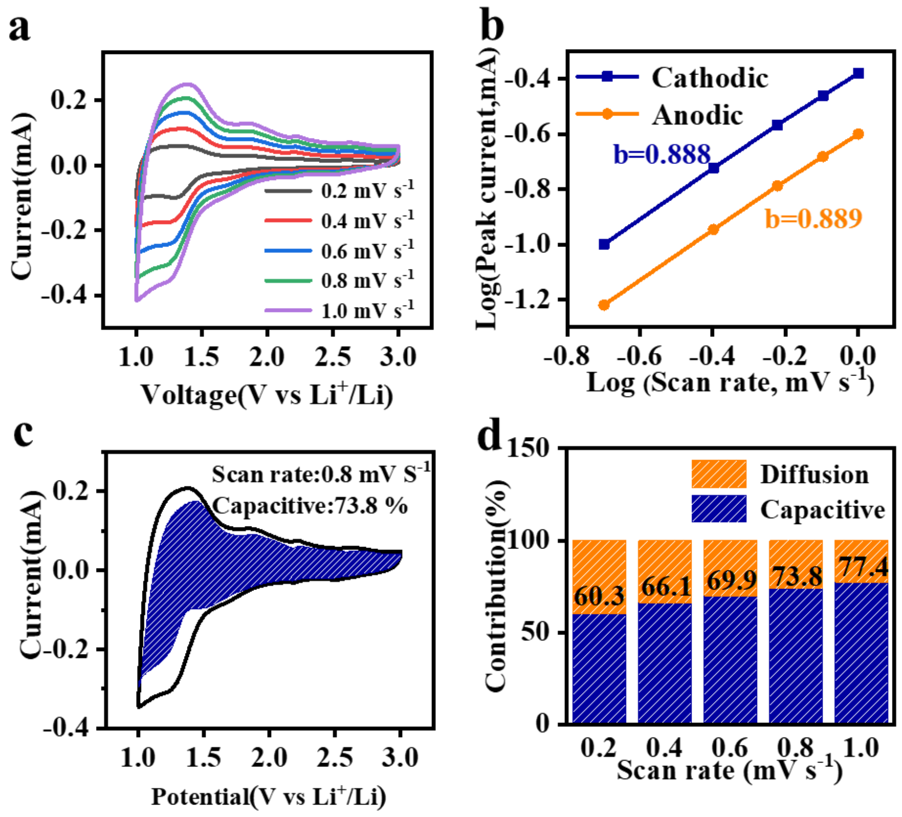

Fig. S8 The charge-storage behavior of $VNbO_4$ anode. **a** CV curves at different sweep rates. **b** Normalized reduction and oxidation peak currents. **c** Pseudo capacitance fitting pattern of all samples at sweep rate of 0.8 mV s^{-1}. **d** Pseudo capacitance contribution

The kinetics of $VNbO_4$ in electrochemical lithium storage can be studied by cyclic voltammetry. Figure S8a shows the CV curves at different sweep rates. The area enclosed by CV curve increasing with the sweep rate, while the voltage values corresponding to peak currents does not change significantly. The following formula was employed to explain the relationship between current values and sweep rates. The i, v values are respectively corresponding to current value (A), sweep rate (mV s^{-1}) while a, b are variable factors.

$$i = av^b \tag{S1}$$

The b value could be obtained to judge the electrochemical behavior of $VNbO_4$-18 after fitting the CV curves at all sweep rate (Figure S8b). Obviously, the obtained b values (0.888 and 0.889) of redox process represent the pseudo capacitive charge storage mechanism controlled by surface of material. In addition, the contribution of pseudo capacitance could be fitted by the following equation. The i and v values are respectively corresponding to current on selected voltage and the sweep rate, while k_1 and k_2 are variable factors.

$$i = k_1 v + k_2 v^{1/2} \tag{S2}$$

The fitting results of CV curve with sweep rate of 0.8 mV s^{-1} and the contribution of pseudo capacitance at different sweep rates are shown in Figure S8c and d, indicating that $VNbO_4$ has a surface-controlled pseudo capacitance behavior kinetics.

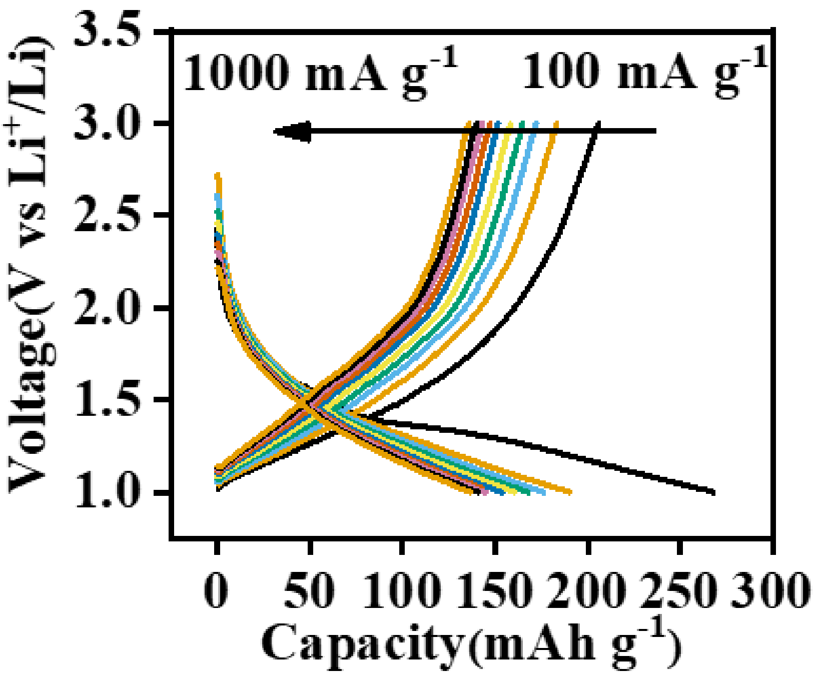

Fig. S9 Charge-discharge profiles of $VNbO_4$ at different current density

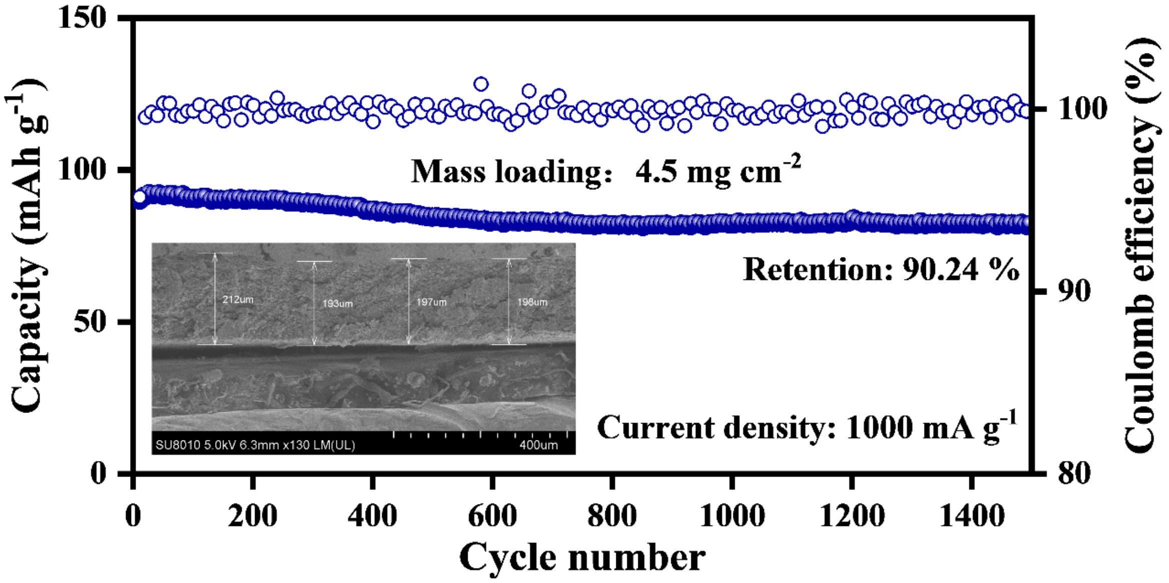

Fig. S10 Cycle performance of $VNbO_4$ electrode at 1.0 A g^{-1} with mass loading of 4.5 mg cm^{-2} (The inset image shows the cross-section of the electrode)

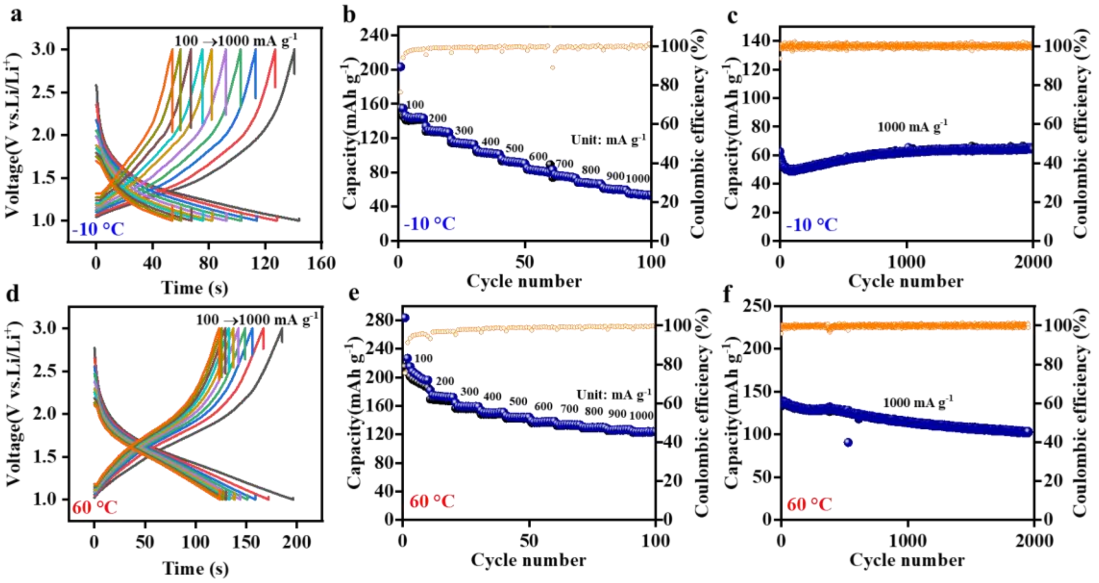

Fig. S11 a Charge-discharge profiles, **b** rate performance of $VNbO_4$ at different current density of $VNbO_4$ at -10 °C. **c** Cycle performance of $VNbO_4$ at current density of 1.0 A g^{-1} at -10 °C. **d** Charge-discharge profiles, **e** rate performance of $VNbO_4$ at different current density of $VNbO_4$ at 60 °C. **f** Cycle performance of $VNbO_4$ at current density of 1.0 A g^{-1} at 60 °C

In order to study the electrochemical performance of $VNbO_4$ in a wide temperature range, constant current charge/discharge tests were carried out at -10 °C and 60 °C, respectively (Figure S11). Figure S11a shows the discharge/charge profiles under different current densities at -10 °C. The result shows that the material experienced more obvious polarization than room temperature, which is due to the slow Li^+ migration kinetics caused by viscous electrolyte at low temperature. The rate performance test shows that $VNbO_4$ delivered the initial charge capacity 159.2 mAh g^{-1} at 100 mA g^{-1} with coulomb efficiency of 77.2 %. When the current density increases to 1000 mA g^{-1}, $VNbO_4$ still delivered a high capacity of 59.2 mAh g^{-1} (Figure S11b). Although the rate capacity had a significant attenuation compared with that at room temperature, which due to the low Li^+ diffusion rate at low temperature as well as the high transfer charge impedance, but it is still satisfactory overall, especially considering the test was carried out at such extreme low temperature conditions. The cycle performance at a current density of 1000 mA g^{-1} is shown in Figure S11c. After 2000 cycles, the capacity retention rate of $VNbO_4$ was 102.1%. This increase in capacity can be attributed to the slow activation process at low temperature. When the electrochemical test was carried out at 60 ° C, the polarization degree of the material did not change significantly compared with that at room temperature (Figure S11d). The rate performance test results show that the initial discharge/charge capacity (283.5 mAh g^{-1} and 221.2 mAh g^{-1}) of the material was higher than that at room temperature, which can be explained by the enhanced Li^+ diffusion ability at high temperature, so that the extra Li^+ could insert in bulk of electrode (Figure S11e). However, the coulomb efficiency of the previous cycles was low, due to the formation of thick SEI film caused

by the violent reaction between electrolyte and material surface at high temperature. At the current density of 1000 mA g^{-1}, the capacity retention rate after 2000 cycles was 77.8 % (Figure S11f). The cycle stability was slightly worsen compared with that at room temperature, which can be explained by the crystal rupture caused by the accumulated stress of excessive lithium insertion. In conclusion, the wide temperature range test results show that $VNbO_4$ still shows good electrochemical characteristics at both -10 °C and 60 °C, indicating that this new material has high application potential at extreme temperatures.

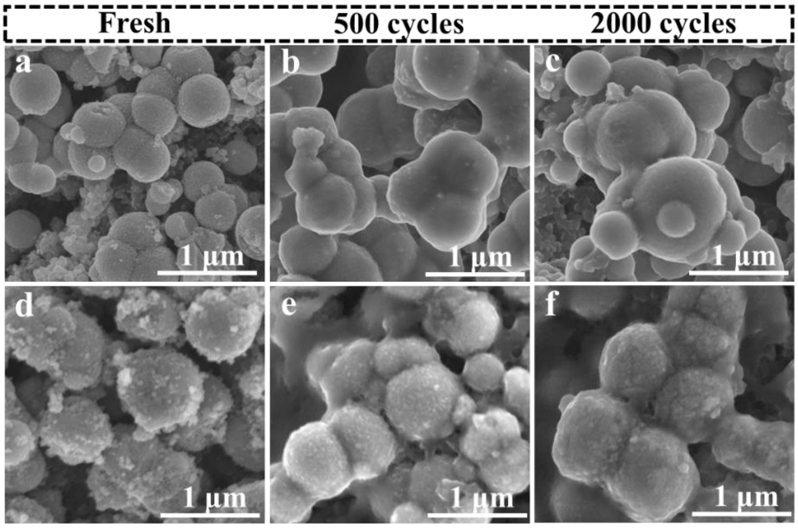

Fig. S12 a-c SEM images of $VNbO_4$ at different cycles. **d-f** SEM images of T-Nb_2O_5 at different cycles

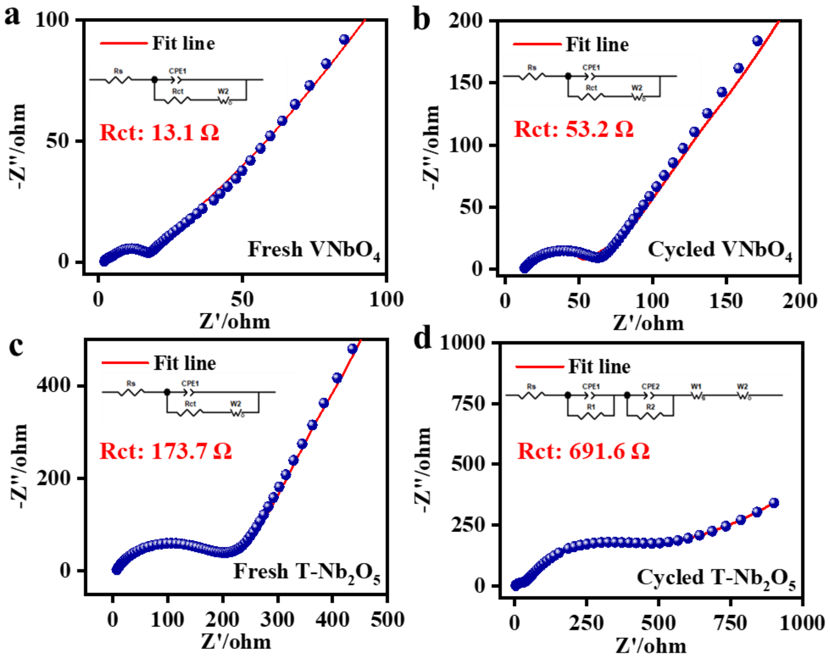

Fig. S13 Nyquist plots and the fitting results of all samples. **a** Fresh $VNbO_4$. **b** Cycled $VNbO_4$. **c** Fresh T-Nb_2O_5. **d** Cycled T-Nb_2O_5

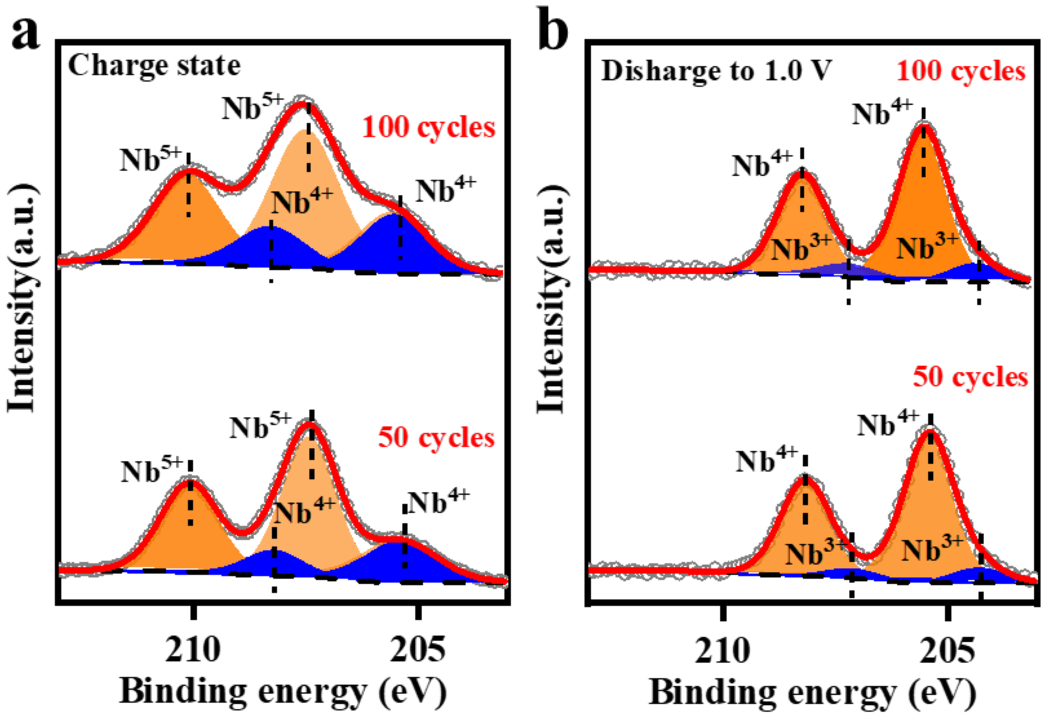

Fig. S14 The Nb 3d orbit XPS spectra of T-Nb_2O_5 electrode at **a** charge state and **b** discharge sate in the 50th and 100th charge-discharge process at 1 A g^{-1}

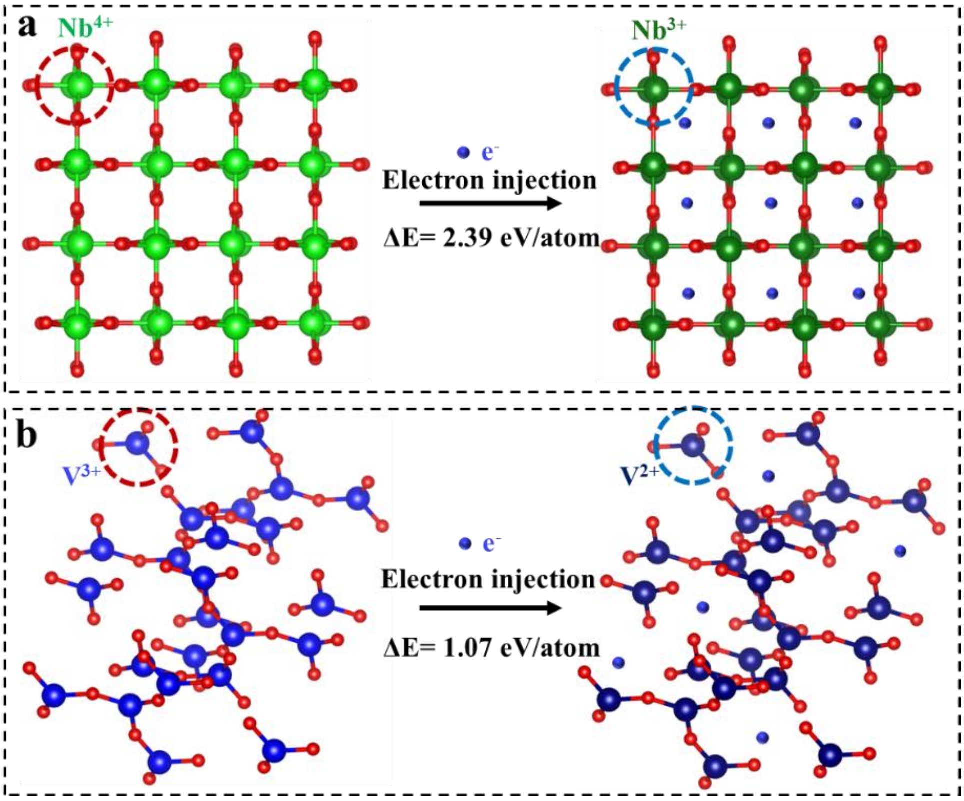

Fig. S15 a Energy changes in the NbO_2 model before and after electron injection. **b** Energy changes in the V_2O_3 model before and after electron injection

Table S2 Li-Nb/Li-V atomic spacing in different models and overall energy difference (normalized based on model 1)

	Model 1	Model 2	Model 3	Model 4
Li-Nb1 distance (Å)	2.98	2.93	2.97	2.95
Li-Nb2 distance (Å)	2.74	4.84	4.89	4.86
Li-Nb3 distance (Å)	2.74	2.76	4.89	4.86
Li-V1 distance (Å)	2.47	2.48	2.47	2.47
Li-V2 distance (Å)	2.47	2.48	2.47	2.47
Li-V3 distance (Å)	3.86	3.55	3.55	3.56
Li-V4 distance (Å)	3.86	3.85	3.55	3.56
Li-Nb average distance (Å)	2.82	3.51	4.25	4.22
Li-V average distance (Å)	3.165	3.09	3.01	3.015
ΔE(eV)	0	-0.19	-0.4	-3.9

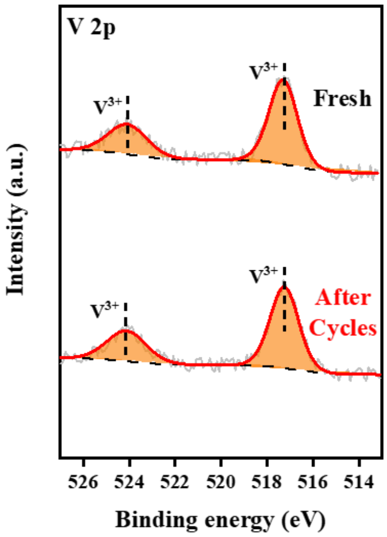

Fig. S16 Post-mortem analysis of V 2p spectra of $VNbO_4$ after 1000 cycles

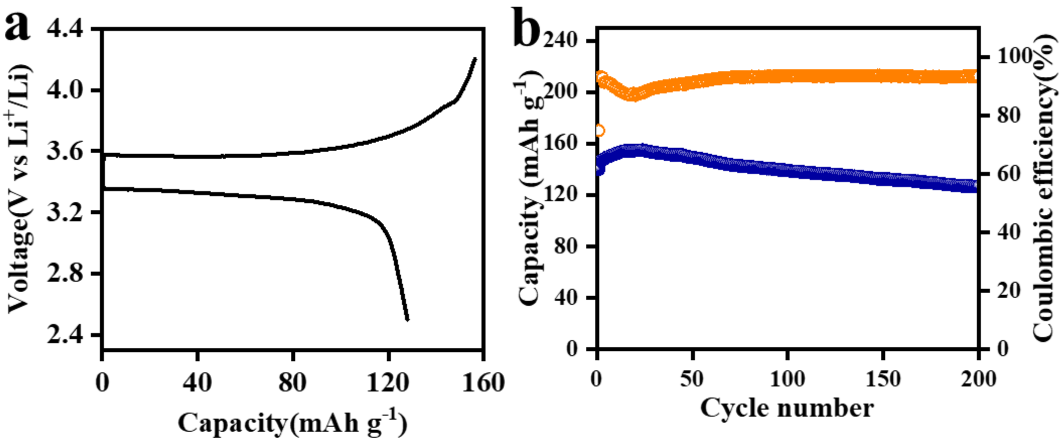

Fig. S17 **a** The initial charge-discharge curves of $LiFePO_4$@C at 1 C, **b** Cycle performance of $LiFePO_4$@C at 1 C

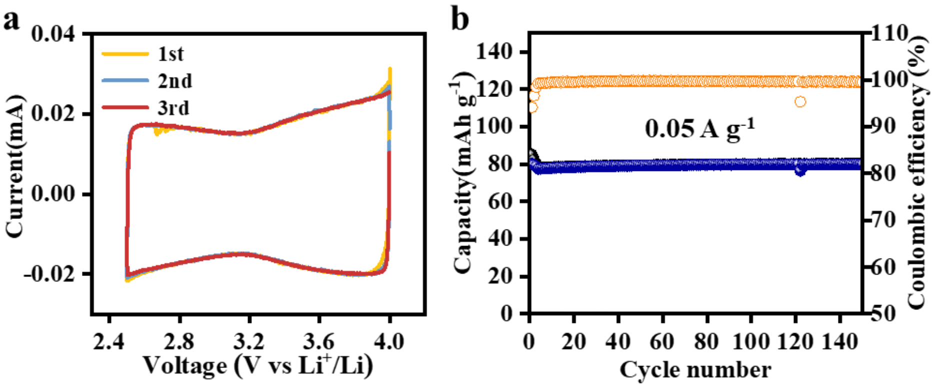

Fig. S18 a The initial three CV curves of AC, **b** Cycle performance of AC at 0.05 A g^{-1}.

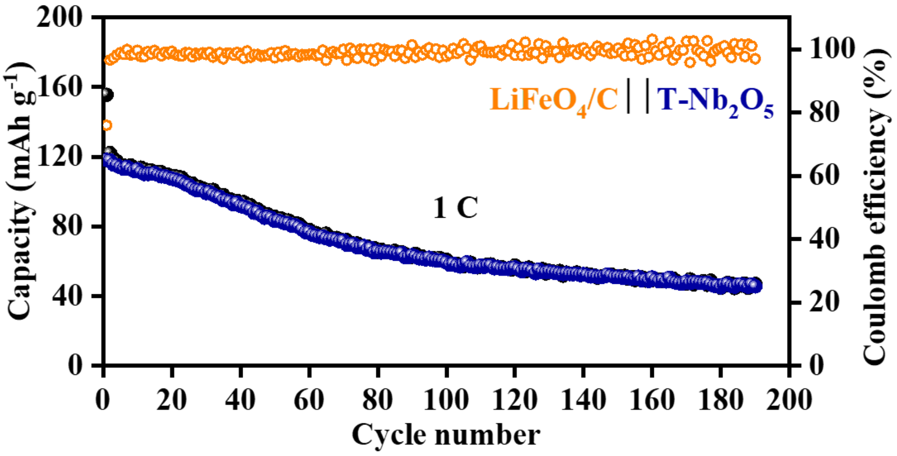

Fig. S19 Cycle performance of $LiFePO_4$@C//T-Nb_2O_5 full cell at 1 C

Supplementary References

[S1] K.J. Griffith, A.C. Forse, J.M. Griffin, C.P. Grey, High-rate intercalation without nanostructuring in metastable Nb2O5 bronze phases. J. Am. Chem. Soc. **138**, 8888 (2016). https://doi.org/10.1021/jacs.6b04345

[S2] S.F. Lou, X.Q. Cheng, L. Wang, J.L. Gao, Q. Li et al., High-rate capability of three-dimensionally ordered macroporous T-Nb2O5 through Li+ intercalation pseudocapacitance. J. Power Sources **361**, 80 (2017). https://doi.org/10.1016/j.jpowsour.2017.06.023

[S3] H.L. Yang, H.H. Xu, L.B. Wang, L. Zhang, Y.H. Huang et al., Microwave-assisted rapid synthesis of self-assembled T-Nb2O5 nanowires for high-energy hybrid supercapacitors. Chem. Eur. J. **23**, 4203 (2017). https://doi.org/10.1002/chem.201700010

[S4] J.M. Zhang, H. Chen, X.W. Sun, X.J. Kang, Y. Zhang et al., High intercalation

pseudocapacitance of free-standing T-Nb2O5 nanowires@carbon cloth hybrid supercapacitor electrodes. J. Electrochem. Soc. **164**, A820 (2017). https://doi.org/10.1149/2.1351704jes

[S5] X.D. Liu, G.Y. Liu, H. Chen, J.M. Ma, R.X. Zhang, Facile synthesis of Nb2O5 nanobelts assembled from nanorods and their applications in lithium ion batteries. J. Phys. Chem. Solids **111**, 8 (2017). https://doi.org/10.1016/j.jpcs.2017.07.007

[S6] D.P. Cao, Z.G. Yao, J.J. Liu, J.C. Zhang, C.L. Li, H-Nb2O5 wired by tetragonal tungsten bronze related domains as high-rate anode for Li-ion batteries. Energy Stor. Mater. **11**, 152 (2018). https://doi.org/10.1016/j.ensm.2017.10.005

[S7] B.H. Deng, T.Y. Lei, W.H. Zhu, L. Xiao, J.P. Liu, In-plane assembled orthorhombic Nb2O5 nanorod films with high-rate Li+ intercalation for high-performance flexible Li-ion capacitors. Adv. Funct. Mater. **28**, 1704330 (2018). https://doi.org/10.1002/adfm.201704330

[S8] Y.Z. Jiao, H.T. Zhang, H.L. Zhang, A. Liu, Y.X. Liu et al., Highly bonded T-Nb2O5/rGO nanohybrids for 4 V quasi-solid state asymmetric supercapacitors with improved electrochemical performance. Nano Res. **11**, 4673 (2018). https://doi.org/10.1007/s12274-018-2049-1

[S9] H.W. Chen, H.X. Zhang, Y.H. Wu, T. Zhang, Y.C. Guo et al., Nanostructured Nb2O5 cathode for high-performance lithium-ion battery with Super-P and graphene compound conductive agents. J. Electroanal. Chem. **827**, 112 (2018). https://doi.org/10.1016/j.jelechem.2018.08.037

[S10] H. Park, D. Lee, T. Song, High capacity monoclinic Nb2O5 and semiconducting NbO2 composite as high-power anode material for Li-Ion batteries. J. Power Sources **414**, 377 (2019). https://doi.org/10.1016/j.jpowsour.2019.01.015

[S11] J.H. Zhang, H.T. Zhang, Y.Q. Zhang, J.W. Zhang, H.Y. He et al., Unveiling of the energy storage mechanisms of multi -modified (Nb2O5@C)/rGO nanoarrays as anode for high voltage supercapacitors with formulated ionic liquid electrolytes. Electrochimica Acta **313**, 532 (2019). https://doi.org/10.1016/j.electacta.2019.04.160

[S12] X.Y. Han, P.A. Russo, N. Goubard-Bretesche, S. Patane, S. Santangelo et al., Exploiting the condensation reactions of acetophenone to engineer carbon-encapsulated Nb2O5 nanocrystals for high-performance Li and Na energy storage systems. Adv. Energy Mater. **9**, 1902813 (2019). https://doi.org/10.1002/aenm.201902813

[S13] S.D. Fu, Q. Yu, Z.H. Liu, P. Hu, Q. Chen et al., Yolk–shell Nb2O5 microspheres as intercalation pseudocapacitive anode materials for high-energy Li-ion capacitors. J. Mater. Chem. A **7**, 11234 (2019). https://doi.org/10.1039/C9TA02342A

[S14] S. Hemmati, G. Li, X.L. Wang, Y.L. Ding, Y. Pei et al., 3D N-doped hybrid architectures assembled from 0D T-Nb2O5 embedded in carbon microtubes toward high-rate Li-ion capacitors. Nano Energy **56**, 118 (2019). https://doi.org/10.1016/j.nanoen.2018.10.048

[S15] Q. Wang, Z.Y. Jia, L.G. Li, J. Wang, G.G. Xu et al., Coupling niobia nanorods with a multicomponent carbon network for high power lithium-ion batteries. Acs Appl. Mater. Interfaces **11**, 44196 (2019). https://doi.org/10.1021/acsami.9b14819

[S16] H.F. Yu, L. Xu, H.Y. Wang, H. Jiang, C.Z. Li, Nanochannel-confined synthesis of Nb2O5/CNTs nanopeapods for ultrastable lithium storage. Electrochimica Acta **295**, 829 (2019). https://doi.org/10.1016/j.electacta.2018.11.017

[S17] Z.H. Song, H. Li, W. Liu, H.Z. Zhang, J.W. Yan et al., Ultrafast and stable Li-(de)intercalation in a large single crystal H-Nb2O5 anode via optimizing the homogeneity of electron and ion transport. Adv. Mater. **32**, 2001001 (2020). https://doi.org/10.1002/adma.202001001

[S18] L. Qin, Y. Liu, S.Y. Xu, S.C. Wang, X. Sun et al., In-plane assembled single-crystalline T-Nb2O5 nanorods derived from few-layered Nb2CTx MXene nanosheets for advanced Li-ion capacitors. Small Methods **4**, 2000630 (2020). https://doi.org/10.1002/smtd.202000630

[S19] Z.Q. Hu, Q. He, Z. Liu, X. Liu, M.S. Qin et al., Facile formation of tetragonal-Nb2O5 microspheres for high-rate and stable lithium storage with high areal capacity. Sci. Bull. **65**, 1154 (2020). https://doi.org/10.1016/j.scib.2020.04.011

[S20] H.R. Li, D. Li, J. Shi, Z.Y. He, Z.C. Zhao et al., Carbon coated 3D Nb2O5 hollow nanospheres with superior performance as an anode for high energy Li-ion capacitors. Sustain. Energy Fuels **4**, 4868 (2020). https://doi.org/10.1039/D0SE00804D

[S21] D.W. Liang, L. Hu, L.L. Wang, L.L. Liu, S. Liang et al., Laser-assisted fabrication of pseudohexagonal phase niobium pentoxide nanopillars for lithium ion battery anodes. Chemnanomat **6**, 73 (2020). https://doi.org/10.1002/cnma.201900532

[S22] P.P. Jing, K.T. Liu, L. Soule, E.H. Wang, T.T. Li et al., Engineering the architecture and oxygen deficiency of T-Nb2O5-carbon-graphene composite for high-rate lithium-ion batteries. Nano Energy **89**, 106398 (2021). https://doi.org/10.1016/j.nanoen.2021.106398

[S23] Y. Chen, Z.Y. Pu, Y.B. Liu, Y.X. Shen, S.M. Liu et al., Enhancing the low-temperature performance in lithium ion batteries of Nb2O5 by combination of W doping and MXene addition. J. Power Sources **515**, 230601 (2021). https://doi.org/10.1016/j.jpowsour.2021.230601

[S24] M. Yang, S. Li, J.G. Huang, Three-dimensional cross-linked Nb2O5

polymorphs derived from cellulose substances: insights into the mechanisms of lithium storage. Acs Appl. Mater. Interfaces **13**, 39501 (2021). https://doi.org/10.1021/acsami.1c11720

[S25] Y.J. Zheng, Z.G. Yao, Z. Shadike, M. Lei, J.J. Liu et al., Defect-concentration-mediated T-Nb2O5 anodes for durable and fast-charging li-ion batteries. Adv. Funct. Mater. **32**, 2107060 (2022). https://doi.org/10.1002/adfm.202107060

[S26] F. Liu, Z. Zhu, Y.G. Chen, J.S. Meng, H. Wang et al., Dense T-Nb2O5/carbon microspheres for ultrafast-(dis)charge and high-loading lithium-ion batteries. Acs Appl. Mater. Interfaces **14**, 49865 (2022). https://doi.org/10.1021/acsami.2c15697

[S27] M.N. Liu, C. Yan, Y.G. Zhang, Separability criteria via sets of mutually unbiased measurements. Sci. Rep. **5**, 8326 (2015). https://doi.org/10.1038/srep13138

[S28] H. Kim, E. Lim, C. Jo, G. Yoon, J. Hwang et al., Ordered-mesoporous Nb2O5/carbon composite as a sodium insertion material. Nano Energy **16**, 62 (2015). https://doi.org/10.1016/j.nanoen.2015.05.015

[S29] X. Wang, C.Y. Yan, J. Yan, A. Sumboja, P.S. Lee, Orthorhombic niobium oxide nanowires for next generation hybrid supercapacitor device. Nano Energy **11**, 765 (2015). https://doi.org/10.1016/j.nanoen.2014.11.020

[S30] L. Wang, X. Zhang, C. Li, Y.N. Xu, Y.B. An et al., Cation-deficient T-Nb2O5/graphene Hybrids synthesized via chemical oxidative etching of MXene for advanced lithium-ion capacitors. Chem. Eng. J. **468**, 143507 (2023). https://doi.org/10.1016/j.cej.2023.143507

[S31] W. Fang, Y. Zhang, C. Kang, Q. Meng, A.R. Shi et al., Oxygen vacancies Nb2O5-x: Ultrastable lithium storage anode materials for advanced rechargeable batteries. Appl. Surf. Sci. **600**, 154068 (2022). https://doi.org/10.1016/j.apsusc.2022.154068

[S32] W.X. Zhang, P.C. Mao, Y.H. Jin, H. Ming, Z.P. Li et al., Oxygen-deficient polymorphic Nb2O5 micro/nanoscale three-dimensionally interconnected anodes with enhanced rate capability for lithium storage. J. Alloys Compd. **911**, 165064 (2022). https://doi.org/10.1016/j.jallcom.2022.165064

[S33] D.W. Kim, S.Y. Kim, K.S. Yang, Synthesis of freestanding binder- and additive-free carbon nanofiber with graphene-wrapped Nb2O5 composite anode for lithium-ion batteries. Nanotechnology **33**, 015602 (2022). https://doi.org/10.1088/1361-6528/ac162d

[S34] J. Lin, S.Y. Zhao, T.G. Tranter, Z.Y. Zhang, F. Peng et al., Modelling and experimental investigation of Nb2O5 as a high-rate battery anode material. Electrochimica Acta **443**, 141983 (2023). https://doi.org/10.1016/j.electacta.2023.141983

[S35] X.B. Ding, H.Y. Huang, Q.H. Huang, B.R. Hu, X.K. Li et al., Doping sites modulation of T-Nb2O5 to achieve ultrafast lithium storage. J. Energy Chem. **77**, 280 (2023). https://doi.org/10.1016/j.jechem.2022.10.049

[S36] G.Y. Liu, S.S. Liu, H. Chen, X.D. Liu, X.W. Luo et al., Highly [001]-oriented N-doped orthorhombic Nb_2O_5 microflowers with intercalation pseudocapacitance for lithium-ion storage. Nanoscale **14**, 11710 (2022). https://doi.org/10.1039/D2NR03187F

[S37] W.X. Zhang, P.X. Shen, L.Z. Qian, Y. Wang, H. Arandiyan et al., Nanoscale phase engineering in two-dimensional niobium pentoxide anodes toward excellent electrochemical lithium storage. Acs Appl. Energy Mater. **4**, 4551 (2021). https://doi.org/10.1021/acsaem.1c00186

[S38] T. Yang, Z.Z. Yang, X.F. Cheng, YH. Ding, Y.X. Fan et al., Three-dimensional hierarchical urchin-like Nb2O5 microspheres wrapped with N-doped carbon: An advanced anode for lithium-ion batteries. J. Alloys Compd. **876**, 160145 (2021). https://doi.org/10.1016/j.jallcom.2021.160145
